# Integrative Analysis of Complement System to Prognosis and Immune Infiltrating in Colon Cancer and Gastric Cancer

**DOI:** 10.3389/fonc.2020.553297

**Published:** 2021-02-03

**Authors:** Dandan Bao, Chenghao Zhang, Longlong Li, Haihong Wang, Qiuyan Li, Leilei Ni, Yinfeng Lin, Rong Huang, Zhangwei Yang, Yan Zhang, Yiren Hu

**Affiliations:** ^1^Department of General Surgery, The Third Clinical Institute Affiliated to Wenzhou Medical University, Wenzhou People’s Hospital, Wenzhou, China; ^2^Emergency department, Wenzhou People’s Hospital, The Third Clinical Institute Affiliated to Wenzhou Medical University, Wenzhou, China; ^3^Department of Gastrointestinal Surgery, People’s Hospital of Deyang City, Sichuan, China; ^4^Department of Oncology, Wenzhou Medical University, Wenzhou, China; ^5^Department of Oncology, The Third Clinical Institute Affiliated to Wenzhou Medical University, Wenzhou People’s Hospital, Wenzhou, China; ^6^Shanghai Institute for Food and Drug Control, Shanghai, China; ^7^Department of Gastroenterology, Yijishan Hospital, the First Affiliated Hospital of Wannan Medical College, Wuhu, China; ^8^Department of General Surgery, Medical College of Soochow University, The Third Clinical Institute Affiliated to Wenzhou Medical University, Wenzhou People’s Hospital, Wenzhou, China

**Keywords:** complement system, tumor immunity, prognosis, TME, colon cancer, gastric cancer

## Abstract

**Background:**

The complement system acts as an integral part of the innate immune response, which acts primarily to remove pathogens and injured cells. Emerging evidence has shown the activation of the immune regulatory function of complements in the tumor microenvironment (TME). We revealed the expression levels of various complements in human cancers and their role in tumor prognosis and immune infiltration.

**Methods:**

The differential expression of complements was explored *via* the Tumor Immune Estimation Resource (TIMER) site and the Oncomine database. To investigate whether these differentially expressed complements have correlation with the prognosis of gastric cancer (GC) and colon cancer, their impact on survival was assessed using the PrognoScan database and Kaplan-Meier plotter. The correlations between complements and tumor immune-infiltrating levels and immune gene markers were statistically explored in TIMER based on Spearman’s correlation coefficients and *p*-values.

**Results:**

In two colon cancer cohorts, an increased expression level of DAF (CD55) has statistically significant correlation with poor disease-free survival (DFS). High C3, CR4, and C5aR1 expression levels were significantly related with poor prognosis in GC patients. In addition, C3, CR4, and C5aR1 expression was positively related to the tumor purity and infiltration levels of multiple immune cells in stomach adenocarcinoma (STAD). Moreover, the expression levels of C3, CR4, and C5aR1 were also strongly correlated with various immune marker sets, such as those of tumor-associated macrophages (TAMs), M1 and M2 macrophages, T cell exhaustion, Tregs, and DCs, in STAD. Additionally, CD55 has positive correlation with few immune cell infiltration levels in colon adenocarcinoma (COAD), but its correlation with immune marker sets was not statistically significant.

**Conclusion:**

These findings confirm the relationship between various complements and tumor prognosis and immune infiltration in colon cancer and GC. CD55 may serve as an indicator on the survival prognosis of patients with colon cancer. Furthermore, as biomarkers for poor prognosis in GC, complements C3, CR4, and C5aR1 may provide potential biological targets for GC immunotherapy.

## Introduction

Gastrointestinal (GI) cancers are among the most common malignancies in the world, including gastric cancer (GC), colorectal cancer (CRC), liver cancer (HCC), biliary tract cancer, and pancreatic cancer. Both GC and CRC are among the top five in terms of morbidity and mortality in our country, making them a major public health concern (1, 2). The complement system consists of more than 50 inherent components and membrane binding receptors and regulators, has traditionally been considered a complex network of proteins that respond rapidly to foreign bodies, triggering inflammatory mediators release and inducing phagocytic reactions and cytolysis (3, 4). A series of membrane-bound proteins, regulatory proteins, cofactors, and receptors are involved in innate immune recognition, adaptive cellular stimulation and proinflammatory responses (5). Three main pathways of complement activation have been described to date: the classical pathway (CP), the alternative pathway (AP), and the Mannose-binding lectin (MBL) pathway. All three pathways converge at the cleavage of C3. Complement activation cleaves C5 into bioactive fragments. C5a is a potent inflammatory mediator that initiates a sequence of protein interactions that meditate complementary dependent cytotoxicity (CDC) by inducing the synthesis of the membrane attack complex (MAC).

A large number of studies in the past decade have provided evidence that activation of complement pathways in the tumor microenvironment (TME) enhances cancer cell proliferation and metastasis either directly or indirectly. For example, C3aR and C5aR agitation increases ovarian cancer cell proliferation (6). In the Lewis lung cancer model, C5a promotes tumor proliferation and growth by creating an immunosuppressive microenvironment (7–9). C5a contributes to the metastasis of colon cancer by up-regulating the expression of IL-10, TGF-β1, Arg-1, and monocyte chemoattractant protein-1 (MCP-1) (10). In addition, MBL deficiency was significantly less frequent among mannan-binding lectin (MBL) patients compared to controls (14 *vs.* 33%). Therefore low levels of MBL may protect against the initiation and progression of GBM (11). Additionally, increased expression of MBL-associated serine protease 2 (MASP-2) predict poor overall survival and tumor recurrence in colon cancer (12). Products of complement activation also play important roles in the immunosuppression of tumor cells, which were achieved by promoting the differentiation of immune cells and up-regulating cytokines such as TG F-β1 and Arg-1, IL-10, PDL-1 (13). Moreover, the membrane-bound complement regulatory proteins (mCRPs), including MCP (CD46), DAF (CD55), and CD59, have been found overexpression in many cancer cells. For example, CD46, CD55, and CD59 were reported to be up-regulated through p-ERK1/2/NF-κB signaling to protect breast cancer from CDC (14). In ovarian cancer, CD46 expression was linked to shorter revival-free time and an overall less favorable prognosis (15). In colon cancer, CD55 serves as a marker of tumor aggression correlated with poor 7-year survival (16). Other cancers that show high expression of CD55 and worse clinical prognoses as a result include prostate cancer, ovarian cancer, AML, CML, ALL, gastric carcinoma, and cervical cancer (17–22). Similarly, increased CD59 expression has been shown to be associated with reduced survival in CRC patients (23), and with reduced overall survival and progression-free survival in patients with prostate adenocarcinoma and diffuse large B cell lymphoma (24, 25) These studies indicate that complements can be used as biomarkers to assess cancer diagnosis and prognosis and have potential application value in tumor immune regulation. The multiple roles of complement intrinsic components and associated regulatory factors of are outlined in **Supplementary Tables 1** and **2**.

In our study, we present an integrative analysis about complements expression and correlation with prognosis and immune infiltration in cancer patients through Oncomine, TIMER (Tumor Immune Estimation Resource), PrognoScan, Kaplan-Meier plotter, and the GEPIA. This present study aims to elucidate the specific role of complements in colon and gastric cancers from a new perspective, and provide a potential biological target for tumor immunotherapy.

## Materials and Methods

### Study Patients

There were a total of 810 cases of GC enrolled in this study, including 566 male patients and 244 female patients. Five hundred twenty cases of Colon cancer including four data sets were included, namely GSE12945 (n = 62), GSE17536 (n = 177), GSE14333 (n = 226), and GSE17537 (n = 55). The correlation analysis of histopathological characteristics and prognosis include gender, TMN stage, Lauren classification, tumor differentiation, and HER2 status. The treatment includes simple surgery and 5FU based adjuvant, which also have impact on survival and prognosis. Survival efficacy evaluation indicators included overall survival (OS), progression-free survival (PFS), and disease-free survival (DFS). OS is the time from randomization to death due to any cause. DFS is defined as the time from randomization to the first tumor recurrence/metastasis or death due to any cause. PFS is the time from the start of randomization to the time of first tumor progression or death. The last follow-up time was the end point for the patients who were lost to follow-up. For patients who were still alive at the end of the study, the end of follow-up was the end point.

### Inclusion and Exclusion Criteria

The inclusion criteria were as follows: (1) Patients in the TCGA, GEO, and EGA databases with a diagnosis of GC or colon cancer and analyzed for RNA sequencing expression. (2) There are clear criteria for the diagnosis and staging of cases. (3) The study data may provide OR (odds ratio)/HR (hazard ratio) and its 95% confidence interval, or can be converted to OR and its 95% confidence interval. The exclusion criteria were as follows: (1) Repeat reported cases. (2) Data is incomplete and survival is unclear. (3) The statistical methods were incorrect and could not be corrected, the OR/HR value and its 95% confidence interval could not be provided, and the measurement data could not provide the mean and standard deviation.

### Differential Expression Analysis

The differential expression of complements in human cancers were explored *via* TIMER (26) and Oncomine (27). TIMER database was applied to identify specific complements or associated regulatory factors that are up- or down-regulated in tumor samples than that in normal tissues by DiffExp module, which were statistically evaluated by Wilcoxon test. To further confirm the results, increased or decreased expression of complements and regulators was explored *via* Oncomine database [the threshold was determined as a fold change of 1.5, gene rank (of all genes), and a p-value of 0.001].

### Survival Analysis

The biological relevance of complements expression to clinical prognosis was evaluated *via* PrognoScan database and Kaplan-Meier plotter. PrognoScan is a publicly available cancer microarray datasets for meta-analysis of the prognostic value (Cox *p*-value < 0.05). Kaplan-Meier plotter covers a database of 21 types of human cancer, obtained from The Gene Expression Omnibus (GEO), European Genome-Phenome Archive (EGA), and Cancer Genome Atlas (TCGA) with a threshold was adjusted by a log-rank *p*-value of <0.05. Moreover, the GEPIA database (28) was used for survival analysis in 33 different cancers using the Mantel-Cox test and the log-rank test.

### Immune Infiltration Analysis

Relevance of complements expression to tumor immune infiltration was analyzed *via* TIMER. The TIMER web server contains homologous data from the TCGA database and is capable of systematically analyzing of immune infiltration levels. The correlation between each complement expression and tumor immune cells infiltrates [i.e., CD8^+^ and CD4^+^ T cells, macrophages, neutrophils, and dendritic cells (DCs)] was estimated by the TIMER algorithm. Relationships between complements and immune gene marker sets were explored in the correlation module. The gene markers of tumor-infiltrating immune cells, including T cells, monocytes, tumor-associated macrophages (TAMs), M1 and M2 macrophages, neutrophils, DCs, Tregs, and T cell exhaustion, were referenced from prior studies (29–31). The correlation module can generate scatter plots of complement and immune-infiltrating genes in specific cancer types, which are statistically evaluated by Spearman’s correlation coefficients and p-values (adjusted by a log-rank p-value <0.05).

### Statistical Analysis

The differential expression of complements in TIMER was explored using the Wilcoxon test. The expression levels of complements generated in Oncomine. Survival was assessed with PrognoScan, K-M plotter and GEPIA, and the curve diagrams are displayed as the Cox p-value and ln (HR) (log-rank test). The strength of correlation was defined according to the following absolute value criteria: 0.00–0.19, very weak; 0.20–0.39, weak; 0.40–0.59, moderate; 0.60–0.79, strong; and 0.80–1.0, very strong. p-values <0.05 were considered statistically significant.

## Results

### The Differential Expression of Complements in Colon and Gastric Cancer

**Figure 1:**
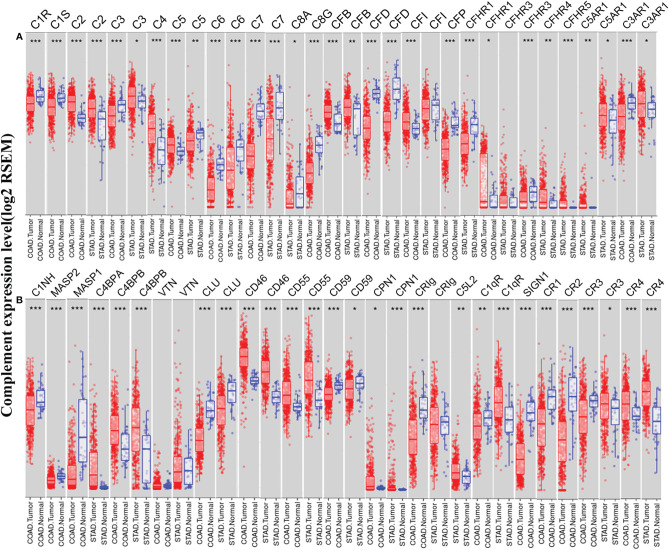
Expression levels of complements in colon adenocarcinoma (COAD) and stomach adenocarcinoma (STAD) in the TIMER database. **(A)** The expression of complement components and some regulators was up- or down-regulated in tumor samples compared with adjacent normal tissues. **(B)** Up- or down-regulated expression of partial complement regulators (***P < 0.001, **P < 0.01, *P < 0.05).

To understand the differences in complement expression between human cancer and normal tissues, complement expression was explored across the TCGA database *via* TIMER. Our results revealed that complement expression is up- or down-regulated in different types of cancer (**Figure 1**). Our study focused on the correlations of complements to prognostic impact and immune infiltration in colon cancer and GC. Compared to normal tissues, the expression of complement proteins, such as C2, C3, C5, CFB, CFI, CFHR1, CR4 (ITGAX), C4BPB, CD46, CD55, and CPN1, was significantly higher in COAD tissues. In contrast, the expression of complement proteins such as C3, C6, C7, C8G, CFD, CFP, CFHR3, CR1, CR2, CR3 (ITGAM), C3aR1, C1INH, MASP2, MASP1, CLU, CD59, CRIg, C1qR, and SIGN1 was lower in COAD tissues. Moreover, compared to normal tissues, in STAD tissues, the expression of C2, C3, C4, CFB, CFHR3, CFHR4, CFHR5, CR3, CR4, C5aR1, C4BPA, C4BPB, CD46, CD55, CPN1, C5L2, and C1qR was higher, and the expression of C5, C6, C7, C8A, CFD, CFP, C3aR1, CLU, CD59, and CRIg was significantly lower.

**Figure 2:**
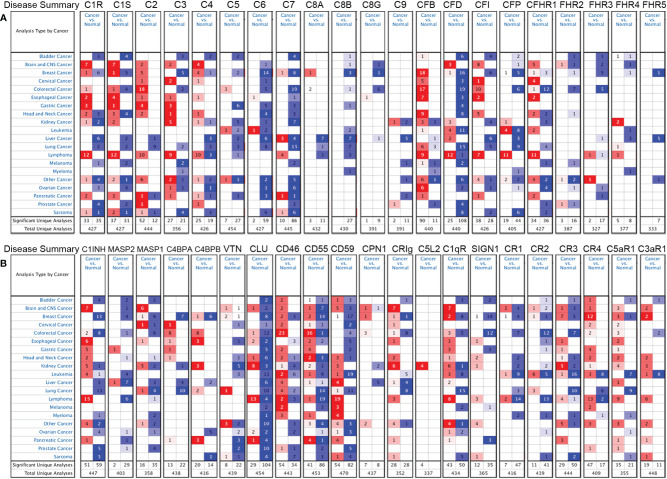
Differential expression of complements in various types of cancer in the Oncomine database. **(A)** The expression of complement components and some regulators up- or down-regulated in tumor samples. **(B)** Increased or decreased expression of partial complement regulators.

We further confirmed the differential gene expression between CRC and GC samples and normal tissues using independent datasets in Oncomine (**Figure 2**). The expression of complements C1R, C1S, C2, C5, CFB, CFI, C8A, C9, CR4 (ITGAX), C5aR1, C1INH, C4BPA, C4BPB, CLU, CD46, CD55, CPN1, CRIg, and C1qR was significantly higher in CRC samples, while the expression of complements C1S, C3, C6, C7, C8B, CFD, CFP, CFHR1, CFHR2, CFHR3, CR1, CR2, CR3 (ITGAM), C3aR1, C1INH, MASP2, MASP1, CLU, CD55, CD59, CRIg, C1qR, and SIGN1 was lower in CRC samples than that in normal tissues. In GC, we found that the expression of C1R, C1S, C2, C3, CR3, CR4, C5aR1, C3aR1, C1INH, MASP2, C4BPA, CD46, CD55, and C1qR was higher than that in normal tissues, whereas the expression of C5, C6, C7, C8B, CFD, CR2, CLU, CD59, and SIGN1 was significantly lower than that in normal tissues.

### Prognostic Analysis of Complements in Colorectal Cancer and Gastric Cancer

**Figure 3:**
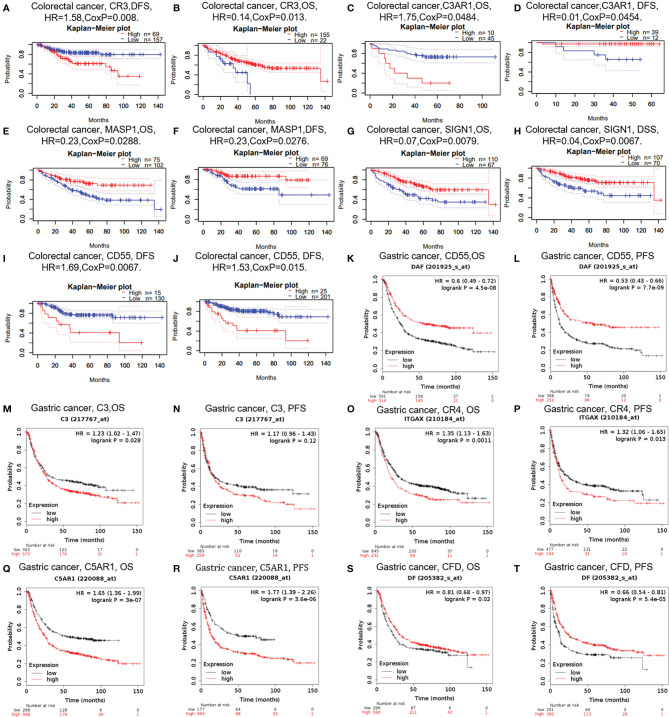
Survival curves of complements generated by PrognoScan **(A–J)** and Kaplan-Meier plotter **(K–T)** for CRC and GC. OS, overall survival; PFS, progression-free survival; DFS, disease-free survival.

In cancer research, the relevance of complements to clinical outcome may suggest the potential pathogenesis of disease and stimulate further researches. The impact of complements on CRC survival was evaluated through the GEPIA and PrognoScan databases. The results showed that three types of complements, namely CR3, C3AR1, and DAF (CD55), were associated with prognosis of CRC patients (**Figures 3A–J**). Two cohorts (GSE14333, GSE17536) of CR3 and two cohorts (GSE17537, GSE12945) of C3AR1 showed no significant association with the prognosis of CRC (**Figures 3A–D**). However, the high expression of MASP1 (OS HR = 0.23, 95% CI = 0.06–0.86, CoxP = 0.0288; DFS HR = 0.23, 95% CI = 0.06–0.85, CoxP = 0.0276) and SIGN1(CD209) (OS HR = 0.07, 95% CI = 0.01–0.51, CoxP = 0.0079; DSS HR = 0.04, 95% CI = 0.00–0.42, CoxP = 0.0067) was associated with a good prognosis in CRC (**Figures 3E–H**). However, two cohorts (GSE17536, GSE14333) of CRC samples showed increased expression of the decay acceleration factor CD55 (DFS HR = 1.69, 95% CI = 1.16–2.48, CoxP = 0.0067; DFS HR = 1.53, 95% CI = 1.09–2.16, CoxP = 0.015) (**Figures 3I, J**), which was significantly correlated with poor survival in CRC patients. Therefore, it is conceivable that CD55 is an independent biomarker that predicts a poor prognosis in patients with CRC.

In addition, we examined the potential effects of complements on GC prognosis *via* the Kaplan-Meier plotter database. Our study revealed that the poor prognosis of GC was related to the high expression of C3 [OS HR= 1.23, 95% CI = 1.02 to 1.47, P = 0.028; progression-free survival (PFS) HR = 1.17, 95% CI = 0.96 to 1.43, P = 0.12), CR4 (ITGAX) (OS HR = 1.35, 95% CI = 1.13 to 1.63, P = 0.0011; PFS HR = 1.32, 95% CI =1.06 to 1.65, P = 0.013), and C5AR1 (OS HR = 1.65, 95% CI = 1.36 to 1.99, P = 3e-07; PFS HR = 1.77, 95% CI =1.39 to 2.26, P =3.6e-06) (**Figures 3M–R**). Furthermore, the increased expression of CFD was related with prolonged OS and PFS in GC (**Figures 3O, P**), but the expression of CD55 did not. These findings revealed that the expression of C3, CR4, and C5AR1 has important significance in the poor prognosis of GC and can be used as prognostic factors. Other detailed results of complement expression are summarized in **Supplementary Figures 1** and **2**.

Based on these results, we confirmed the prognostic value of the complement system in cancer. The results are summarized as follows: High CD55 expression is significantly associated with poor prognosis in CRC, and MASP1 and SIGN1 expression is associated with a good prognosis in CRC. In addition, the increased expression of C3, CR4, and C5aR1 is correlated with a poor prognosis in GC. However, the increased expression of CFD is correlated with prolonged OS and PFS in GC.

### Correlation of C3, CR4, CD55 Expression and Prognosis With Different Clinicopathological Factors in Gastric Cancer Patients

**Figure 4:**
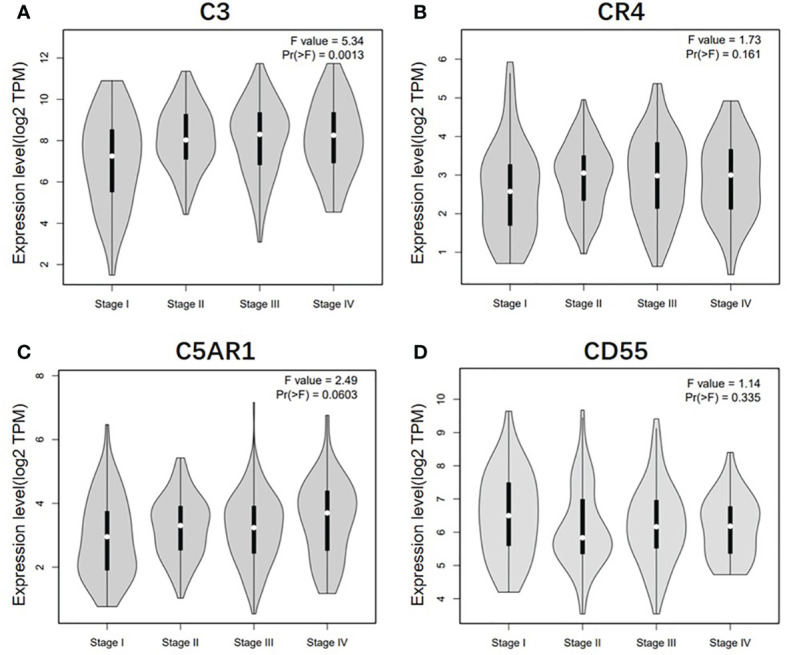
Correlation of C3, CR4, C5AR1, and CD55 expression level with pathological stage of GC and CRC patients. **(A)** Pathological stage plot of C3 expression in GC patients. **(B)** Pathological stage plot of CR4 expression in GC patients. **(C)** Pathological stage plot of C5AR1 expression in GC patients. **(D)** Pathological stage plot of CD55 expression in CRC patients.

To further understand the relevance of complement expression in cancer, we used TCGA database to study the relationship between complement expression and clinical characteristics *via* Kaplan-Meier plotter. Increased expression of C3, CR4, and C5aR1 lead to worse OS and PFS was associated with gender, Lauren classification and differentiation (*P* < 0.05). In addition, high C3, CR4, and C5aR1 expression was correlated with worse OS and PFS in stage N1/N1-N3 of GC, but has no correlation with patients in stage N0 (**Table 1**). The correlation of complements expression level of C3, CR4, CD55 with pathological stage of GC and CRC was shown in **Figure 4**. This feature generates expression violin plots based on patient pathological stage. Here the stage T describes the size or direct extent of the primary tumor. High C3 expression level has correlation with worse OS and PFS in stage T2 (OS HR = 2.1, P = 0.0012; PFS HR = 1.79, P = 0.0051), and increased expression of C5AR1 resulted in the highest HR values of OS and PFS in T4 stage (OS HR = 2.28, P = 0.05; PFS HR = 2.77, P = 0.011). In addition, N category refers to lymph node involvement, N0 indicates tumor cells absent from regional lymph nodes, and N1–N3 indicate regional lymph node metastasis present. It is worth noting that in the N staging table, increased C3, CR4, and C5aR1 expression has the highest HR values for GC patients with N1 stage. These results suggested that C3, CR4 and C5aR1 expression level can impact the prognosis in gastric cancer patient with depth of tumor invasion and lymph node metastasis.

**Table 1:** Correlation of C3, CR4, and C5aR1 expression and clinical prognosis in gastric cancer with different clinicopathological factors *via* Kaplan-Meier plotter.

		C3				CR4(ITGAX)				C5aR1			
Clinicopathological characteristics	N	Overall survival (n = 881)		Progression-free survival (n = 645)		Overall survival (n = 881)		Progression-free survival (n = 645)		Overall survival (n = 881)		Progression-free survival (n = 645)	
		Hazard ratio	*P-*value	Hazard ratio	*P-*value	Hazard ratio	*P-*value	Hazard ratio	*P-*value	Hazard ratio	*P-*value	Hazard ratio	*P-*value
**SEX**													
Female	244	1.59	**0.017**	1.6	**0.022**	1.45	0.054	1.6	**0.016**	1.72	**0.0025**	1.65	**0.0087**
Male	566	1.19	0.11	1.16	0.24	1.36	**0.009**	1.3	**0.04**	1.98	**7.3e-07**	1.95	**7.7e-06**
**STAGE**													
1	69	2.45	0.22	0.47	0.17	0.33	**0.024**	0.41	0.097	0.41	0.092	0.26	**0.02**
2	145	2.17	**0.0094**	1.77	0.06	0.77	0.4	1.3	0.41	2.3	**0.007**	2.25	**0.011**
3	319	1.75	**0.0022**	1.86	**0.0043**	1.32	0.069	0.79	0.24	1.75	**0.00046**	1.73	**0.0035**
4	152	1.37	0.11	1.32	0.19	0.74	0.13	1.51	0.074	1.76	**0.0038**	1.48	0.051
STAGE T													
2	253	2.1	**0.0012**	1.79	**0.0051**	1.26	0.32	1.19	0.41	1.52	**0.05**	1.52	**0.046**
3	208	1.23	0.28	1.19	0.3	0.73	0.11	0.81	0.26	1.75	**0.0026**	1.52	**0.022**
4	39	1.63	0.26	1.82	0.13	0.69	0.4	1.83	0.13	2.28	**0.05**	2.77	**0.011**
STAGE N													
0	76	1.43	0.41	1.44	0.39	0.51	0.11	0.52	0.12	0.5	0.13	0.54	0.17
1	232	2.17	**0.0002**	2.06	**0.00022**	1.7	**0.017**	1.73	**0.0075**	2.36	**2.8e-05**	2.34	**6.2e-05**
2	129	2	**0.0067**	1.84	**0.013**	0.59	0.055	0.67	0.13	2.43	9.3e-05	2.37	8.2e-05
3	76	1.74	**0.042**	1.73	**0.042**	0.75	0.3	0.7	0.18	1.82	**0.027**	1.54	0.14
1+2+3	437	1.75	**2.3e-05**	1.71	**2.6e-05**	1.14	0.33	1.17	0.25	2.1	**1.6e-08**	1.85	**1.4e-06**
STAGE M													
0	459	1.68	**2e-04**	1.62	**0.00034**	1.17	0.33	1.26	0.14	1.79	**3.4e-5**	1.63	**0.0003**
1	58	2.18	**0.022**	2.09	**0.035**	0.55	**0.046**	0.49	**0.019**	2.58	**0.0031**	1.48	0.21
LAUREN CLASSIFICATION													
Intestinal	336	1.73	**0.0015**	1.69	**0.0039**	1.74	9e-04	1.71	**0.0027**	2.58	**2.2e-8**	2.13	**2.7e-05**
Diffuse	248	1.6	**0.009**	1.76	**0.0093**	0.63	**0.014**	0.61	**0.0084**	1.46	**0.029**	1.37	0.092
DIFFERENTIATION													
Poor	166	0.69	0.072	0.63	0.069	0.79	0.27	0.71	0.14	0.78	0.3	1.26	0.33
Moderate	67	1.39	0.34	1.42	0.27	3.65	**6.8e-05**	4.23	**3.7e-06**	1.73	0.1	1.87	0.051
High	32	4.21	**0.037**	/	/	2.63	**0.029**	/	/	2.91	**0.0126**	/	/
TREATMENT													
Surgery alone	393	1.52	**0.0042**	1.48	**0.0053**	1.15	0.35	1.26	0.17	1.29	0.081	1.33	0.09
5 FU based adjuvant	157	0.52	**0.00032**	0.55	**0.011**	1.59	**0.013**	1.43	0.052	0.78	0.15	0.74	0.085
**HER2 STATUS**													
HER2 negative	641	1.26	**0.043**	1.23	0.12	1.27	0.053	0.81	0.14	1.55	**0.00038**	1.6	**0.004**
HER2 positive	424	1.27	0.11	1.21	0.31	1.57	**0.0015**	2.11	**1e-05**	1.78	**0.00038**	2.45	**2.6e-05**

Bold values indicate P < 0.05.

### Correlation Analysis Between Complement Expression and Immune Infiltration Level in Colon Adenocarcinoma and Stomach Adenocarcinoma

The tumor-infiltrating lymphocyte (TIL) grade is a critical factor for tumor stage and one of the most powerful predictors of cancer recurrence and survival (32, 33). Tumor purity is a crucial index affecting the analysis of TIL infiltration levels (34). To investigate the impact of the expression of complement components on tumor immune infiltration levels, the TIMER web server was used, which contains most of the cognate data of the TCGA (26, 28).

**Figure 5:**
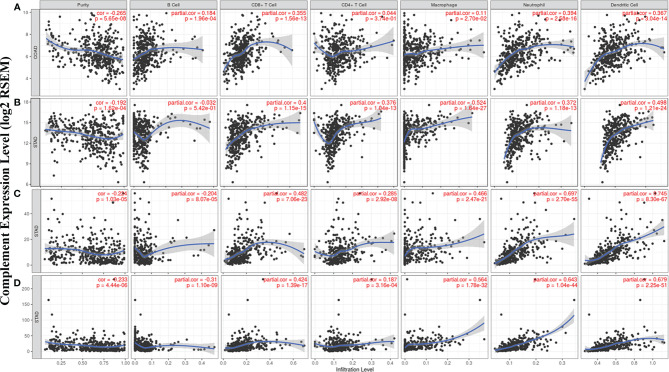
Correlation of CD55, C3, CR4, and C5AR1 expression with immune infiltration levels in COAD or STAD. **(A)** CD55 expression relate to immune cell infiltration levels in COAD (n = 457). **(B)** C3 expression relate to immune cell infiltration levels in STAD (n = 415). **(C)** CR4 expression relate to immune cell infiltration levels in STAD (n = 415). **(D)** C5AR1 expression relate to immune cell infiltration levels in STAD (n = 415).

According to the TIMER database results, we identified some specific complement types, of which their expression levels have significant relevance with clinical prognosis and tumor purity in STAD and/or COAD. The detailed results were shown in Supplementary material (**Figure 3**). Specifically, CD55 expression has significant correlation with tumor purity in COAD, and the expression of the complement component C3, complement receptor CR4, and complement activation regulator C5aR1 was statistically related with immune purity in STAD (**Figure 5**). The specific relationship is described as follows. In COAD, CD55 expression has significant positive relationship with the immune infiltrating levels of CD8^+^ cells (r = 0.355, P = 1.56e-13), neutrophils (r = 0.394, P = 2.58e-16), and DCs (r = 0.367, P = 3.04e-14) but no significant correlation with the infiltrating levels of CD4^+^ T cells, B cells, and macrophages (**Figure 5A**). In STAD, C3 expression has moderate positive correlations with the immune infiltrating levels of CD8^+^ cells (r = 0.4, P = 1.15e-15), CD4^+^ T cells (r = 0.376, P = 1.04e-13), macrophages (r = 0.524, P = 1.64e-27), neutrophils (r = 0.372, P = 1.18e-13) and DCs (r = 0.498, P = 1.21e-24) but not B cells (**Figure 5B**). CR4 expression has moderate to strong positive correlations with the immune infiltrating levels of CD8^+^ cells (r = 0.482, P = 7.06e-23), macrophages (r = 0.466, P = 2.47e-21), neutrophils (r = 0.697, P = 2.70e-55), and DCs (r = 0.745, P = 8.30e-67), and a weak correlation with the immune-infiltrating level of CD4^+^ T cells (r = 0.285, P = 2.92e-08) but not B cells in STAD (**Figure 5C**). C5AR1 expression has moderate to strong positive correlations with the infiltrating levels of CD8^+^ cells (r = 0.424, P = 1.39e-17), macrophages (r = 0.564, P = 1.78e-32), neutrophils (r = 0.643, P = 1.04e-44), and DCs (r = 0.679, P = 2.25e-51) but not B cells and CD4^+^ T cells in STAD (**Figure 5D**). These results strongly suggest that the complements CD55, C3, CR4, and C5AR1 play important roles in the regulation of immune infiltration in COAD and STAD.

### Correlation Between Complement Expression and Immune Marker Sets

Given the relationship of complements with immune-infiltrating levels in COAD and STAD and the potential mechanism of immune interactions, we further studied the correlations between complements and marker genes in tumor-infiltrating immune cells, including T cells, TAMs, M1 and M2 macrophages, monocytes, T cell exhaustion, Tregs, and DCs, from COAD and/or STAD tissues. Previous studies have demonstrated that DCs promote cancer metastasis by reducing CD8^+^ T cell cytotoxicity and increasing Tregs (35, 36). FOXP3 has important value in suppressing killing ability of cytotoxic T cells on target tumor cells (8, 37).

**Figure 6:**
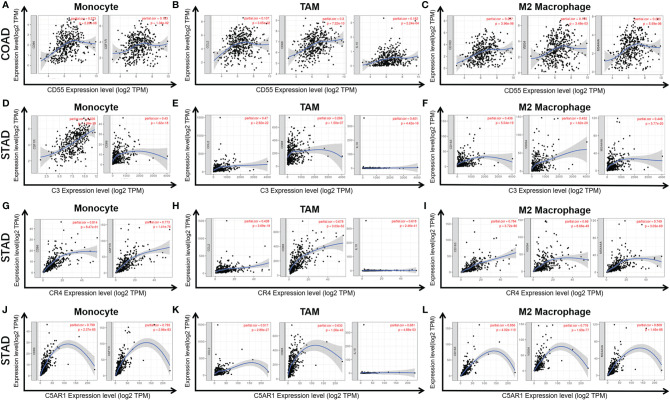
Correlation of CD55 **(A–C)**, C3 **(D–F)**, CR4 **(G–I)**, and C5AR1 **(J–L)** expression with macrophage polarization in COAD or STAD. Immune gene markers include those of monocytes (CSF1R, CD86), tumor-associated macrophages (TAMs) (IL10, CD68, and CCL2) and M2 macrophages (MS4A4A, CD163 and VSIG4).

CD55 expression has moderate positive correlations with the infiltrating levels of CD8^+^ cells, neutrophils and DCs. After adjusting the correlations by purity, we found that the relevance of CD55 expression to most sets of gene markers, such as those of monocytes (CSF1R), TAMs (CCL-2, IL10), M2 macrophages (MS4A4A, CD163, and VSIG4), Tregs (FOXP3, CCR8, TGFβ1, and STAT5B), DCs (HLA-DPB1, CD1C, NRP1, and ITGAX), and T cell exhaustion (TIM-3, LAG3, CTLA4, and PD-1) were not statistically significant (**Table 2**, **Figures 6A–C**). Therefore, evidence for a correlation between CD55 and immune cells is insufficient, and the mechanism by which CD55 acts in tumor immunity remains unclear.

**Table 2:** Correlation analysis between CR3, CR4, C5aR1, CD55 and relate immune genes markers dependently in STAD and COAD *via* TIMER.

Description	Gene markers	C3(STAD)				CR4 (STAD)				C5aR1 (STAD)				CD55 (COAD)			
		None		Purity		None		Purity		None		Purity		None		Purity	
		Cor	*P*	Cor	*P*	Cor	*P*	Cor	*P*	Cor	*P*	Cor	*P*	Cor	*P*	Cor	*P*
T cell (general)	CD3D	0.444	***	0.402	***	0.518	***	0.477	***	0.432	***	0.382	***	0.245	***	0.149	2.56e-03
	CD3E	0.491	***	0.457	***	0.505	***	0.464	***	0.399	***	0.356	***	0.273	***	0.179	2.84e-04
	CD2	0.482	***	0.451	***	0.577	***	0.549	***	0.478	***	0.45	***	0.285	***	0.2	4.89e-05
Monocyte	CD86	0.442	***	0.396	***	0.809	***	0.796	***	0.809	***	0.799	***	0.359	***	0.273	***
	CD115 (CSF1R)	0.551	***	0.519	***	0.749	***	0.743	***	0.799	***	0.793	***	0.229	***	0.123	1.34e-02
TAM	CCL2	0.461	***	0.429	***	0.457	***	0.394	***	0.554	***	0.517	***	0.203	1.2e-05	0.107	3.05e-02
	CD68	0.259	***	0.222	***	0.632	***	0.608	***	0.648	***	0.632	***	0,363	***	0.3	***
	IL10	0.411	***	0.383	***	0.623	***	0.581	***	0.703	***	0.681	***	0.248	***	0.182	2.24e-04
M1 Macrophage	INOS (NOS2)	−0.024	0.62	−0.047	0.366	0.167	6.67e-04	0.151	3.22e-03	0.141	0.225	0.123	0.199	-0.041	7.23e-01	-0.068	5.65e-01
	IRF5	0.3	***	0.266	***	0.343	***	0.324	***	0.347	***	0.344	***	0.138	2.24e-01	0.155	1.91e-01
	COX2(PTGS2)	0.045	0.359	0.02	0.694	0.125	1.07e-02	0.096	6.21e-02	0.139	***	0.304	***	0.101	3.74e-01	0.132	2.64e-01
M2 Macrophage	CD163	0.459	***	0.42	***	0.754	***	0.743	***	0.863	***	0.856	***	0.128	2.59e-01	0.157	1.85e-01
	VSIG4	0.442	***	0.421	***	0.646	***	0.629	***	0.783	***	0.776	***	0.098	3.88e-01	0.128	2.81e-01
	MS4A4A	0.455	***	0.419	***	0.74	***	0.724	***	0.815	***	0.808	***	0.109	3.37e-01	0.167	1.58e-01
Dendritic cell	HLA-DPB1	0.482	***	0.441	***	0.586	***	0.542	***	0.572	***	0.543	***	0.143	2.08e-02	0.207	7.85e-02
	HLA-DQB1	0.312	***	0.258	***	0.52	***	0.474	***	0.44	***	0.401	***	0.154	1.75e-01	1.279	1.29e-01
	HLA-DRA	0.406	***	0.367	***	0.626	***	0.504	***	0.581	***	0.563	***	0.085	4.53e-01	0.133	2.61e-01
	HLA-DPA1	0.45	***	0.413	***	0.602	***	0.571	***	0.58	***	0.56	***	0.161	1.56e-01	0.218	6.93e-02
	BDCA-1(CD1C)	0.462	***	0.421	***	0.44	***	0.291	***	0.352	***	0.307	***	0.139	2.23e-01	0.205	8.14e-02
	BDCA-4(NRP1)	0.474	***	0.432	***	0.535	***	0.505	***	0.661	***	0.64	***	0.097	3.95e-01	0.077	5.18e-01
	CD11c(ITGAX)	0.447	***	0.409	***	/	/	/	/	0.803	***	0.788	***	0.065	5.67e-01	0.05	6.74e-01
Treg	FOXP3	0.488	***	0.453	0.488	0.591	***	0.567	***	0.49	***	0.469	***	0.043	7.07e-01	0.023	8,45e-01
	CCR8	0.458	***	0.436	0.458	0.678	***	0.683	***	0.615	***	0.614	***	0.148	1.94e-01	0.171	1.48e-01
	STAT5B	0.424	***	0.407	0.424	0.356	***	0.36	***	0.452	***	0.464	***	0.246	2.88e-02	0.243	3.82e-01
	TGFβ (TGFB1)	0.498	***	0.467	0.498	0.419	***	0.384	***	0.5	0.011	-0.233	***	0.061	5.91e-01	0.097	4.15e-01
T cell exhaustion	PD-1 (PDCD1)	0.377	***	0.346	0.377	0.485	***	0.458	***	0.381	***	0.359	***	0.067	5.59e-01	0.152	1.99e-01
	CTLA4	0.332	***	0.294	0.332	0.502	***	0.469	***	0.409	***	0.382	***	0.105	3.59e-01	0.142	2.32e-01
	LAG3	0.336	***	0.309	0.336	0.465	***	0.442	***	0.412	***	0.384	***	-0.063	5.83e-01	-0.087	4.62e-01
	TIM-3(HAVCR2)	0.44	***	0.404	0.44	0.814	***	0.806	***	0.806	***	0.8	***	0.157	1.66e-01	0.197	9.55e-02
	GZMB	0.209	***	0.161	0.209	0.41	***	0.367	***	0.375	***	0.339	***	-0.045	6.94e-01	-0.025	8.37e-01

STAD, stomach adenocarcinoma; Tumor, correlation analysis in tumor tissue of TCGA; Normal, correlation analysis in normal tissue of TCGA. *P < 0.01; **P < 0.001; ***P < 0.0001.

C3 expression in STAD has moderate to strong correlations with the expression levels of gene marker sets of TAMs, macrophages, and monocytes. More specifically, in STAD, marker sets of monocytes (CD86, CSF1R), TAMs (CCL-2, IL10), and M2 macrophages (MS4A4A, CD163 and VSIG4) have significant correlation with C3 expression. In addition, immune marker sets of DCs (HLA-DPB1, CD1C, NRP1, and ITGAX), Tregs (FOXP3, CCR8, TGFβ1, and STAT5B), and T cell exhaustion (TIM-3, LAG3, CTLA4 and PD-1) were also significantly correlated with C3 expression (**Table 2**, **Figures 6D–F**).

We also assessed the correlations of CR4 expression (ITGAX, CD11c, αXβ2 integrin) with tumor immune infiltration levels in GC, and the results were roughly the same. CR4 expression has a significant correlation with most immune markers of TAMs, macrophages, and monocytes, Tregs, DCs and T cell exhaustion in STAD (**Table 2**, **Figures 6G–I**).

In STAD, C5AR1 expression also has significant positive correlations with the immune-infiltrating levels of CD8^+^ T cells, neutrophils, macrophages, and DCs. Most immune marker sets of TAMs, macrophages, monocytes, DCs, Tregs, and T cell exhaustion also has significant relevance with C5AR1 expression in STAD (**Table 2**, **Figures 6J–L**).

These results verified that the complement component C3, complement receptor CR4 and regulator of complement activation C5aR1 play important roles in the regulation of immune infiltration in GC. Therefore, it is conceivable that C3, CR4 and C5AR1 can be recognized as potential immune biomarkers that regulate immune escape in the GC microenvironment.

## Discussion

Complement-associated proteins play complex and important roles in tumor immune regulation. As an essential piece of innate immunity system, the complements also directly or indirectly affects tumorigenesis, development, and metastasis by regulating the functions of macrophages and lymphocytes (38, 39). Recent researches have illustrated the role of various inherent complements and their regulators in antitumor immunity. Complements are associated with a variety of diseases, such as CRC, lung cancer, neuroblastoma, chronic lymphocytic leukemia, ovarian cancer, and gastrointestinal tumors (40, 41). Here, we report that high expression levels of some complement components and regulatory proteins are associated with a poor prognosis in GC and CRC. Moreover, our findings show correlations of tumor immune infiltration levels with the expression of specific types of complements in CRC and GC. In conclusion, the present study further verified a critical role of complement system in tumor microenvironment, suggesting its potential to be a new target for cancer immunotherapy.

In our study, we first examined the differential expression of various complement components and receptor/regulatory proteins compared with normal tissues in the Oncomine and TCGA datasets. In COAD tissues, we found the expression of C2, C5, CFB, CFI, CR4, C4BPB, CD46, CD55, and CPN1 was significantly higher than that in normal tissues. Meanwhile, in STAD tissues, the expression of C2, C3, C4, CR3, CR4, C5aR1, C4BPA, CD46, CD55, and C1qR was significantly higher than that in normal tissues (**Figures 1** and **2**). Second, based on the survival analysis, we found that specific types of complements with differential expression in CRC and GC were also significantly associated with tumor prognosis. The PrognoScan showed that increased CD55 expression level is connected with a high hazard ratio for DFS in CRC patients (**Figures 3I, J**), Depending on the TCGA database examined with Kaplan-Meier plotter, increased C3, CR4, and C5aR1 expression was related to a poor prognosis in GC (**Figures 3M–R**). Another important aspect of our research is the correlation analysis of complement expression to the immune infiltration levels of immune cells in COAD and STAD. Our results revealed significant relevance between multiple complements expression and immune cell infiltration level. Specifically, C3, CR4, and C5AR1 expression has moderate to strong positive relevance with the immune-infiltrating levels of CD8^+^ T cells, neutrophils, macrophages, and DCs in STAD. Moreover, in COAD, the expression of the complement regulatory factor CD55 is positively related with the infiltration levels of CD8^+^ T cells, neutrophils, and DCs (**Figure 5**). These findings undoubtedly suggested the diagnostic and prognostic value of complements in GC and CRC patients, and implicated its potential mechanism in tumor immunoregulation.

Moreover, we further assessed the relevance between complements and immune marker genes to clarify the mechanism of complements in immune regulation in cancers. Most marker sets of monocytes (CD86, CSF1R), TAMs (CCL-2, IL10), and M2 macrophages (MS4A4A, CD163, and VSIG4) were significantly associated with the expression levels of C3, CR4, and C5aR1 in STAD. Furthermore, in STAD, the increased expression of C3, CR4, and C5aR1 was positively correlated with Tregs (FOXP3, CCR8, TGFβ1, and STAT5B) and T cell exhaustion (TIM-3, LAG3, CTLA4, and PD-1) (**Table 2**, **Figure 6**). However, the relevance between CD55 expression and immune markers of TAMs, M2 macrophages, DCs, and T cell exhaustion were not statistically significant. Our results reveal the underlying mechanism of C3, CR4, and C5aR1 in regulating the polarization of tumor-associated macrophages (TAMs) in STAD.

TAMs, myeloid-derived suppressor cells (MDSCs), and tumor-associated neutrophils (TANs) are immunosuppressive cell populations that most infiltrate the TME (42). It has been reported that TAMs contribute to cancer cell progression (43). Cancer cell-derived C3a regulates metabolism and immune activity of TAMs through the C3a receptor-PI3Kγ signaling pathway (44). Activated complement C3 bioactive fragments (C3b, iC3b, and C3dg) recruit macrophages and activate immune cells by binding to receptors CR1 (CD35), CR3 (ITGAM), CR4 (CD11c/ITGAX), and/or VSIG4 (3), thus inducing phagocytosis and modulating the function of antigen-presenting cells. C5a is an active fragment of complement component C5, mediates polarization of macrophages by activating C5a receptor (C5aR) expression and the nuclear factor-κB signaling on TAMs (45). Numerous studies have shown that C5a/C5aR1 activation pathway implicated in the a variety of inflammatory processes and immune diseases pathogenesis (46–48). MDSCs are precursors of DCs, macrophages and/or granulocytes, has the ability to significantly inhibit the immune responses and regulate the polarization of immune cells in the TME (49). MDSCs can inhibit the function of T cells by upregulating programmed cell death 1 ligand (PDL1) through the C5a/C5aR pathway, resulting in suppression of the antitumor immune response (50, 51). In addition, extensive studies have shown that mCRPs are up-regulated in various cancers, of which contains MCP (CD46), DAF (CD55), and CD59 (14, 52). Specifically, mCRPs promote the binding of C1q active fragment to apoptotic cells, recruit factor H (fH) and amplify CP activation, which protects tumor cells from necrotic lysis and inflammation (53). It has been demonstrated *in vivo* that complement inhibitory proteins (DAF, CD59) play important regulatory roles in the development of T cell immunity (3, 54, 55). CD55 is one of glycosyl-phosphatidyl-inositol (GPI)-anchored membrane proteins, and its main role is to accelerate the decay of C3 invertase in the CP and AP (56). Recent new studies have shown that CD55 can induce chemoresistance in tumors by blocking the induction of ICOSL^+^ B cells and has been proposed as an attractive therapeutic target for immunotherapy (57). CD59 has been shown to down-regulate the activity of CD4^+^ T cells (58). Blocking or neutralizing mCRPs in tumor cells has been shown to improve the efficacy of cellular immunotherapy (59, 60).

Interleukin (IL)-10 as a typical anti-inflammatory cytokine, its role in the tumorigenesis and progression is still highly controversial. Multiple studies have found that IL-10 levels is positively correlated with poor prognosis in patients with lung cancer (61), melanoma (62, 63), and T/NK-cell lymphoma (64). Some other studies have also showed that IL-10 can be produced by tumor cells themselves (61, 65, 66), and indicating that its expression is an escape mechanism from immune surveillance (65, 67, 68). It is commonly known that monocytes/macrophages contribute directly to tumor progression by releasing factors that promote angiogenesis and metastasis (69, 70), and certain types of activated macrophages have been shown to produce IL-10 at inflammatory sites (71, 72). Notably, M2-activated macrophages, which are considered to be the majority of Tumor-associated macrophages (TAMs) within a tumor, release significant amounts of IL-10, which could indicate a correlative relationship between IL-10 and tumor progression (73). Nevertheless, there is ample evidence to support that IL-10 has potent anti-tumor effects as well. A possible mechanism that has been proposed is IL-10-mediated stimulation of NK cells (74–77). Mocellin et al. (78) showed that NK cells serve to “link” adaptive immunity to innate immunity might be a key step in tumor immune escape (78). In addition, it is proposed by Tanja Bedke et al. (79) that a combination of several factors, such as the tissue microenvironment, cell types that respond to IL-10 and the cellular source of IL-10 re involved in the dual effects (79). However, how these versatile functions of IL-10 are regulated is still not clearly understood.

Recent studies have indicated that activation of complement promotes cancer (9, 38, 51, 80), and complement inhibitors, as a new strategy for the treatment of multiple malignant tumors, have been gradually reported in clinical application (81–83). Daniel Ajon et al. (81). showed that systemic blocking of C5aR and C3aR signaling could enhance the efficacy of anti–PD-L1/PD-L1 (81, 82). They used syngeneic models of lung cancer, demonstrate that the combination of C5a and PD-1 blockade can significantly reduce tumor progression and metastasis and leads to prolonged survival (81). Haoran Zha et al. (82) used MX53 (a C5aR antagonist) to pharmacologically block C5aR signaling in mice tumor model and also observed a greatly enhance of anti-PD-1/PD-L1 efficacy (82). In addition, Haoran Zha et al. (44) described a mechanism for tumor cell–derived C3 in suppressing antitumor immunity, suggest that tumor cell–derived C3 can be a useful target for cancer immunotherapy (44).

Moreover, a great deal of data showed that mCRP expression, contains CD46, CD55, and CD59, are linked to worse clinical outcomes, and in some cases highly specific for tumor cells, many approaches to block mCRP expression on tumor cells have been studied (17, 22, 84). Anti PD-1/PD-L1 monoclonal antibodies (mAbs), as checkpoint inhibitors, have been employed concomitantly with chemotherapeutic drugs to achieve improved outcomes. Mamidi et al. showed that inhibition of mCRP expression, sensitizes cancerous leukemia cells to complement attack, resulting in enhance d effectiveness of rituximab (85). Similarly, the use of mAbs blocking CD55 and CD59 in addition to Rituximab treatment leads to increased tumor toxicity in non-Hodgkin’s lymphoma (86). In addition, neutralization of CD55 has led to increased complement activation and complement-mediated killing in breast cancer (87), melanoma (56), Burkitt lymphoma (88), and leukemia (89). CD55 has been identified as a signaling protein responsible for self-renewal and therapeutic resistance to cisplatin in endometroid tumors, and blockade of CD55 using saracatinib sensitizes chemo-resistant cells to cisplatin (90). These approaches undoubtedly provided safer and more effective anti-cancer therapeutics, but further clinical studies are needed.

In summary, imbalanced complement activation is the key mechanism of tumor promotion, of which improper regulation leads to the production of activated fragments and the binding of corresponding receptors on the cell surface (5, 91, 92). CD55 may be a potential biomarker for a poor prognosis in patients with colon cancer. High C3, CR4, and C5aR1 expression is related with a poor prognosis and positive immune infiltration levels of immune cells, as well as immune gene markers of M1 and M2 macrophages, TAMs, Tregs, and T cell exhaustion. Therefore, as biomarkers for a poor prognosis in GC, complements C3, CR4, and C5aR1 are expected to provide potential biological targets for GC immunotherapy.

## Data Availability Statement

The datasets presented in this study can be found in online repositories. The names of the repository/repositories and accession number(s) can be found in the article/**Supplementary Material**.

## Author Contributions

DB designed the project. LL, ZY, YZ, HW, QL, YL, and LN participated in the data analysis. BD and CZ interpreted the data and wrote the manuscript. YH provided advice for the project and reviewed the manuscript. YH supervised the project. All authors contributed to the article and approved the submitted version.

## Funding

This work was supported by the National Nature Science Foundation of China (Youth Program Nos. 81702388 for YRH).

## Conflict of Interest

The authors declare that the research was conducted in the absence of any commercial or financial relationships that could be construed as a potential conflict of interest.
