# Supplementary material for: Integrative Analysis of Complement System to Prognosis and Immune Infiltrating in Colon Cancer and Gastric Cancer
Source: Front Oncol. 2021 Feb 3;10:553297. doi: 10.3389/fonc.2020.553297 (PMC7886994; doi:10.3389/fonc.2020.553297)
Supplement: Supplementary file 1 [file DataSheet_1.docx]

**Supplementary Material**

**Supplementary Table 1|** Effects of complement system on the TME and their therapeutic potential for cancer treatment.

| **protein** | **Main expression** | **Connection with disease** | **Functions** **in the TME** | Refs. |
| --- | --- | --- | --- | --- |
| C1r | liver | Periodontal Ehlers-Danlos syndrome | Part of the C1 complex; recognizes surface-bound IgG, IgM, CRP, and PAMP.; initiates CP (classical pathway) | [[1](#_ENREF_1)] |
| C1s | liver | Autoimmune hemolytic anemia | Part of the C1 complex; cleaves C2 and C4 | [[2](#_ENREF_2), [3](#_ENREF_3)] |
| C1q | gland, spleen | Melanoma, cervical cancer, breast cancer, pancreatic cancer, colon cancer and lung cancer, glomerulopathy. | Part of the C1 complex; recognizes surface-bound IgG, IgM, CRP, and PAMP.; Activation of CP | [[2](#_ENREF_2), [4-6](#_ENREF_4)] |
| C2 | liver | SLE (Systemic lupus erythematosus) | Part of the CP/LP convertases; Activated C1 cleaves C2 into C2a and C2b; cleaves C3/C5 | [[7](#_ENREF_7)] |
| C3 | liver | Melanoma, lung cancer, gastric cancer, colon cancer, breast cancer, pancreatic cancer | Activate complement system promote tumor growth, metastasis, EMT and angiogenesis; Progenitor for anaphylatoxin C3a, opsonin C3b and signaling fragments (iC3b, C3c, C3d); part of the AP, C3 and all C5 convertases | [[8-10](#_ENREF_8)] |
| C4 | liver | Oral squamous cell carcinoma, lung cancer | Part of the classical activation pathway; proteolytically cleaved into a trimer of alpha, beta, and gamma chains prior to secretion. The alpha chain is cleaved to release C4 anaphylatoxin, an antimicrobial peptide and a mediator of local inflammation. | [[11](#_ENREF_11), [12](#_ENREF_12)] |
| C5a | liver, lung | Lung cancer, gastric cancer, hepatocellular carcinoma, colorectal cancer, breast cancer, ovarian cancer, melanoma, ovarian cancer, cervical cancer | Promote tumorigenesis, tumor growth, angio-genesis, cell motility and invasiveness and inhibit immune function by inducing MDSCs or decreasing CD8+ T cells. Blockade of C5aR significantly reduced MDSCs and the immunomodulators ARG1, CTLA-4, IL-6, IL-10, LAG3, and PDL-1 | [[13-17](#_ENREF_13)] |
| C6 | liver | Glioma brain cancer | Part of the membrane attack complex that can be incorporated into the cell membrane and cause cell lysis. | [[18](#_ENREF_18)] |
| C7 | Adrenal | Liver cancer | encodes a serum glycoprotein that forms a membrane attack complex together with complement components C5b, C6, C8, and C9 as part of the terminal complement pathway of the innate immune system; Promote the stemness of liver cancer cells | [[19](#_ENREF_19)] |
| C8 | liver | ND | C8 is one component of the membrane attack complex, which mediates cell lysis, and it initiates membrane penetration of the complex. | [[20](#_ENREF_20), [21](#_ENREF_21)] |
| C9 | liver | Age-related macular degeneration (AMD), Hepatitis C virus (HCV) | The final component of the complement system, participates in the formation of the membrane attack complex (MAC); forms lytic pore. | [[22](#_ENREF_22), [23](#_ENREF_23)] |

C, complement component; AP, alternative pathway; CP, classical pathway; LP, lectin pathway; PAMP, (pathogen associated molecular pattern; MDSCs, myeloid-derived suppressor cell; ND, not determined; SLE, systemic lupus erythematosus; AMD, age-related macular degeneration; MAC, membrane attack complex.

**Supplementary Table 2|** Regulators of complement activation.

| **Regulators** | **Gene** | **Main expression** | **Function in the TME** | **Connection with disease** | **Refs.** |
| --- | --- | --- | --- | --- | --- |
| ***Soluble regulators and effectors*** | |  |  |  |  |
| C1 inhibitor (C1INH) | SERPING1 | Liver | Regulates the CP and LP; Blocks serine protease and is a suicide substrate for C1r, C1s, MASP2, coagulation factors and C3b | Hereditary angioedema. | [[24](#_ENREF_24), [25](#_ENREF_25)] |
| MBL-MASP |  |  |  |  |  |
| sMAP (MAp19) | MASP2 | Liver | Form complexes with MBL. Binds to MBL/ Ficolins; cleaves C2 and C4 | ND | [[26](#_ENREF_26)] |
| MAP1 (MAp44) | MASP1 | Liver | Form complexes with MBL and ficolins. Reported to inhibit deposition of C4, may cleave MASP-2 and C3; activates pro-FD | Mutations are associated with 3MC syndrome. | [[27](#_ENREF_27), [28](#_ENREF_28)] |
| C4b-binding protein (C4BP) | C4BPA, C4BPB | Liver | Accelerates decay of CP and LP convertases. Cofactor for FI. Reported to bind apoptotic and necrotic cells and amyloid protein, preventing inflammation. | Mutations associated with aHUS and recurrent pregnancy loss. | [[29](#_ENREF_29), [30](#_ENREF_30)] |
| Factor H(FH) | CFH | Liver | Main regulator of the AP. Accelerates decay of AP convertases. Cofactor for FI. Recognizes self-surfaces; accelerates convertase decay; cofactor for fI. | Gene variants associated with aHUS, AMD and C3G. | [[31-36](#_ENREF_31)] |
| FHR1 (Reconectin) | CFHR1 | Liver | Accelerates convertase decay. Cofactor for FI. | Gene variants associated with aHUS, AMD and C3G | [[37](#_ENREF_37), [38](#_ENREF_38)] |
| FB | CFB | Liver | Part of the AP C3/C5 convertases; cleaves C3/C5 | glioblastoma multiforme, Cutaneous Squamous Cell Carcinoma, pancreatic cancers; | [[39](#_ENREF_39), [40](#_ENREF_40)] |
| Factor I (FI) | CFI | Liver | Protease that mediates degradation of C3b and C4b fragments, interrupting complement cascade activation. | Gene variants associated with aHUS and AMD. Deficiency associated with increased susceptibility to infections. | [[36](#_ENREF_36), [41](#_ENREF_41), [42](#_ENREF_42)] |
| Vitronectin (S-protein, S40) | VTN | Liver | Binds to C5b–C9; prevents assembly of TCC. Also implicated in cell adhesion via binding to integrins | nasopharyngeal carcinoma, gastric cancer | [[43](#_ENREF_43)] |
| Clusterin (Apolipoprotein J, SP-40) | CLU | Reproductive organs | Binds to C7–C9; prevents assembly of TCC, stress-activated chaperone associated with various, physiologic processes. | ND | [[44](#_ENREF_44)] |
| ***Surface bound regulators and effectors*** | | |  |  |  |
| mCRPs |  |  | Protect cancer cells from MAC-mediated CDC and regulate the response of T cells |  |  |
| MCP (CD46) | CD46 | All nucleated cells | Cofactor for FI | Gene variants associated with aHUS. Deficiency associated with male infertility. | [[41](#_ENREF_41), [45](#_ENREF_45)] |
| DAF (CD55) | CD55 | Wide expression | Involved in the regulation of the complement cascade, accelerates convertase decay. | Deficiency associated with cervical cancer, pancreatic cancer; angiopathic thrombosis and protein-losing enteropathy | [[39](#_ENREF_39), [46-48](#_ENREF_46)] |
| CD59 (Protectin) | CD59 | Wide expression | Involved in lymphocyte signal transduction. Binds to C8 and C9, inhibiting the incorporation of multiple copies of C9 into the complex. | Deficiency associated cerebral infarction; PNH; chronic hemolysis and childhood relapsing immune-mediated polyneuropathy; esophageal squamous cell carcinoma | [[49-52](#_ENREF_49)] |
| Carboxypeptidase N (Polypeptide 1) | CPN1 | Liver | Degrades C3a and C5a to their desArg forms. | May cause angioedema, chronic urticaria, asthma, breast Cancer. | [[53-55](#_ENREF_53)] |
| CR1 | CD35 | Appendix, spleen | Binds C3b/iC3b; Induces phagocytosis: accelerates decay of AP, CP and LP convertases; cofactor for fI. | Variants associated with susceptibility to SLE and resistance to malaria. Deficiency associated with glomerulonephritis. Low levels of CR1 on erythrocytes associated with poor outcome in PNH patients;late-onset Alzheimer's disease (LOAD). | [[56-59](#_ENREF_56)] |
| CR2 | CD21 | lymph node, spleen | Binds iC3b/C3d; lowers threshold for B-cell stimulation | Variations associated with susceptibility to systemic lupus erythematosus type 9 (SLEB9). | [[60](#_ENREF_60), [61](#_ENREF_61)] |
| CR3 | MAC1,CD11b/D18, αMβ2 integrin | Liver | Induces phagocytosis via interaction with iC3b; modulates IL-12 family in APC | Variations associated with Keratinocyte Skin Carcinomas, SLE | [[62](#_ENREF_62), [63](#_ENREF_63)] |
| CR4 | CD11C,αXβ2 integrin | Spleen, appendix | iC3b-mediated phagocytosis and inhibition of alternative pathway activation | Variations associated with Triple-negative Breast Cancer (TNBC), chronic lymphocytic leukemia (CLL), Behçet's disease;High expression indicates favorable prognosis in gastric cancer. | [[64-67](#_ENREF_64)] |
| CRIg | VSIG4 | Placenta, lung | Induces phagocytosis via interaction with iC3b/C3c; regulatory effect on C5 convertases | Variations associated with ovarian Cancer, a potential diagnostic marker of severe preeclampsia. | [[68-70](#_ENREF_68)] |
| ***Receptors for complement effector proteins*** | | |  |  |  |
| C5L2(C5aR2, GPF77,) | GPR77 | spleen, appendix | Immune cell recruitment and inflammation and possibly acts as a decoy receptor. | involved in coronary artery disease and in the pathogenesis of sepsis. | [[71-74](#_ENREF_71)] |
| C3aR | None | appendix, placenta | Binds C3a; triggers proinflammatory signaling, an immune Checkpoint Receptor | ovarian cancer cells, melanoma, | [[75](#_ENREF_75), [76](#_ENREF_76)] |
| C5AR | CD88 | appendix, bone marrow | Binds C5a; triggers proinflammatory signaling, an immune Checkpoint Receptor | ovarian cancer, Lewis lung cancer, melanoma, promotes acute pyelonephritis | [[71](#_ENREF_71), [73](#_ENREF_73), [74](#_ENREF_74), [76-78](#_ENREF_76)] |
| C1qR | CD93 | Placenta,lung | Phagocytosis and cell adhesion, link and remodel transmembrane proteins to the cytoskeleton | associated with psoriasis, allergic asthma; also as a Potential target in AMD | [[79-81](#_ENREF_79)] |
| DC-SIGN1 | CD209 | placenta, lymph node | pathogen recognition receptor and a cell adhesion receptor; signalling, inflammation and phagocytosis | associated with decreased susceptibility to pulmonary tuberculosis, Promoting host dissemination and infection, Behçet's disease severity | [[82-84](#_ENREF_82)] |

Abbreviations: AP, alternative pathway; CP, classical pathway; LP, lectin pathway; MASP, MBL-associated serine protease; 3MC syndrome, a rare developmental disorder that unifies the overlapping autosomal recessive disorders previously known as Mingarelli, Malpuech, Michels and Carnevale syndromes; aHUS, atypical haemolytic uraemic syndrome; AMD, age-related macular degeneration; C3G,Cyanidin 3 Glucoside; SLE, systemic lupus erythematosus; PNH, paroxysmal nocturnal haemoglobinuria; TCC, terminal complement complex; CRPs, membrane-bound complement regulatory proteins; MCP, membrane cofactor protein; DAF, decay accelerator factor; PHN, paroxysmal nocturnal hemoglobinuria; ND, not determined; CR1, complement receptor 1;FH, Factor H; FI, Factor I; C4BP, C4b-binding protein; C5aR1, C5a receptor (CD88); SIGNR1, a mouse homologue of DC-SIGN, SIGN-related 1; TCC, terminal complement complex

**Supplementary Figure 1.** Correlation of complements expression with prognostic value in COAD (colon adenocarcinoma).


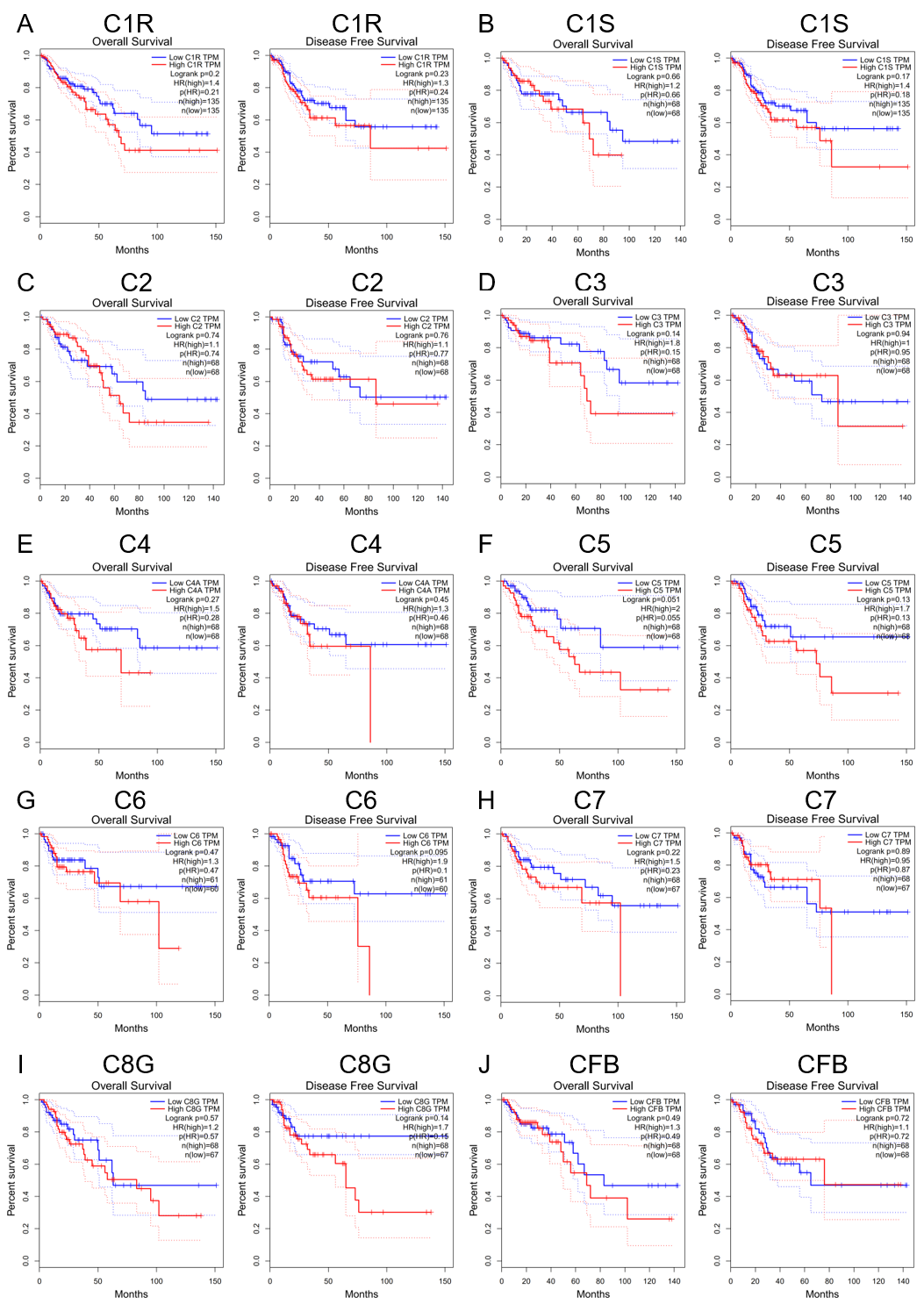

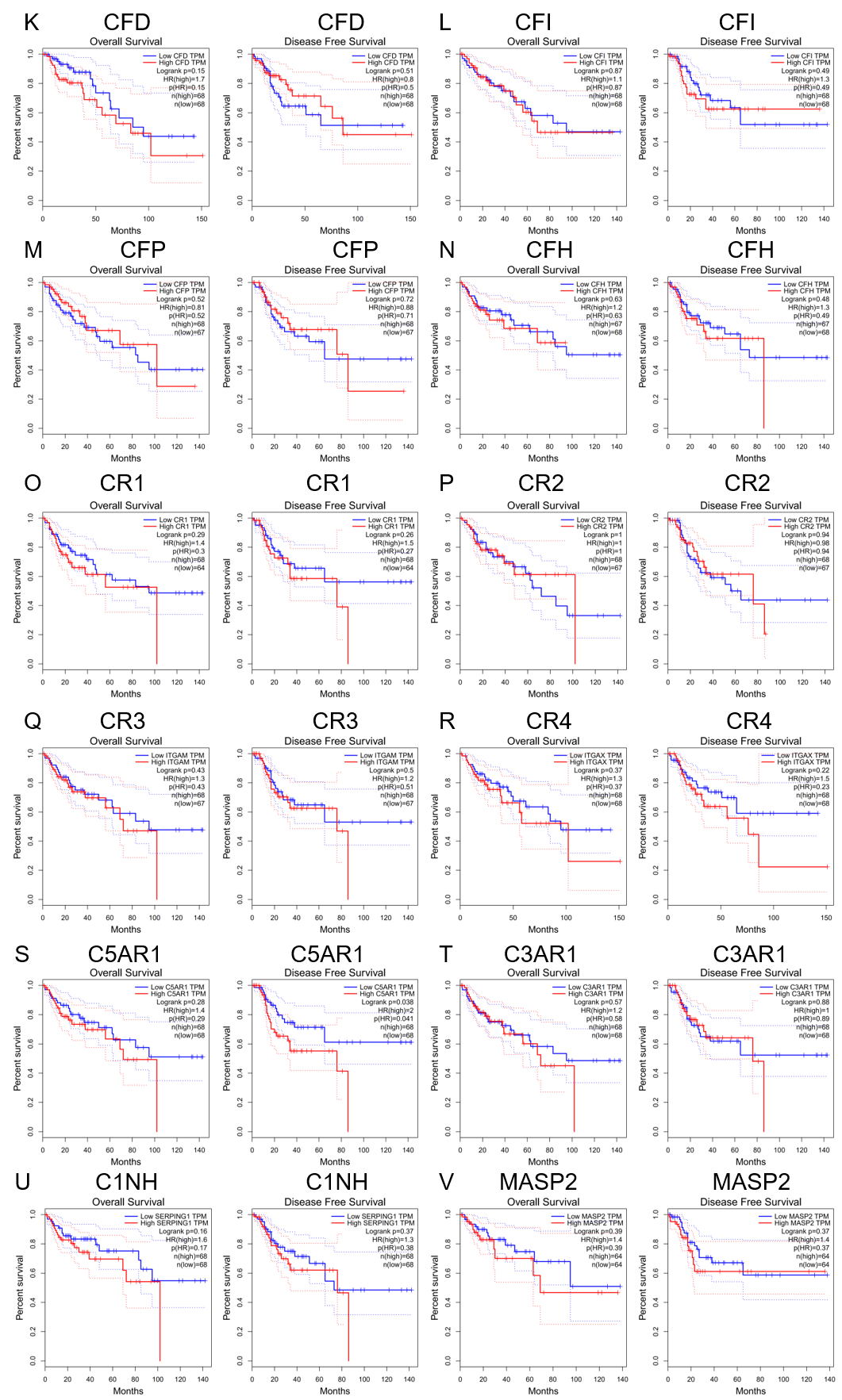

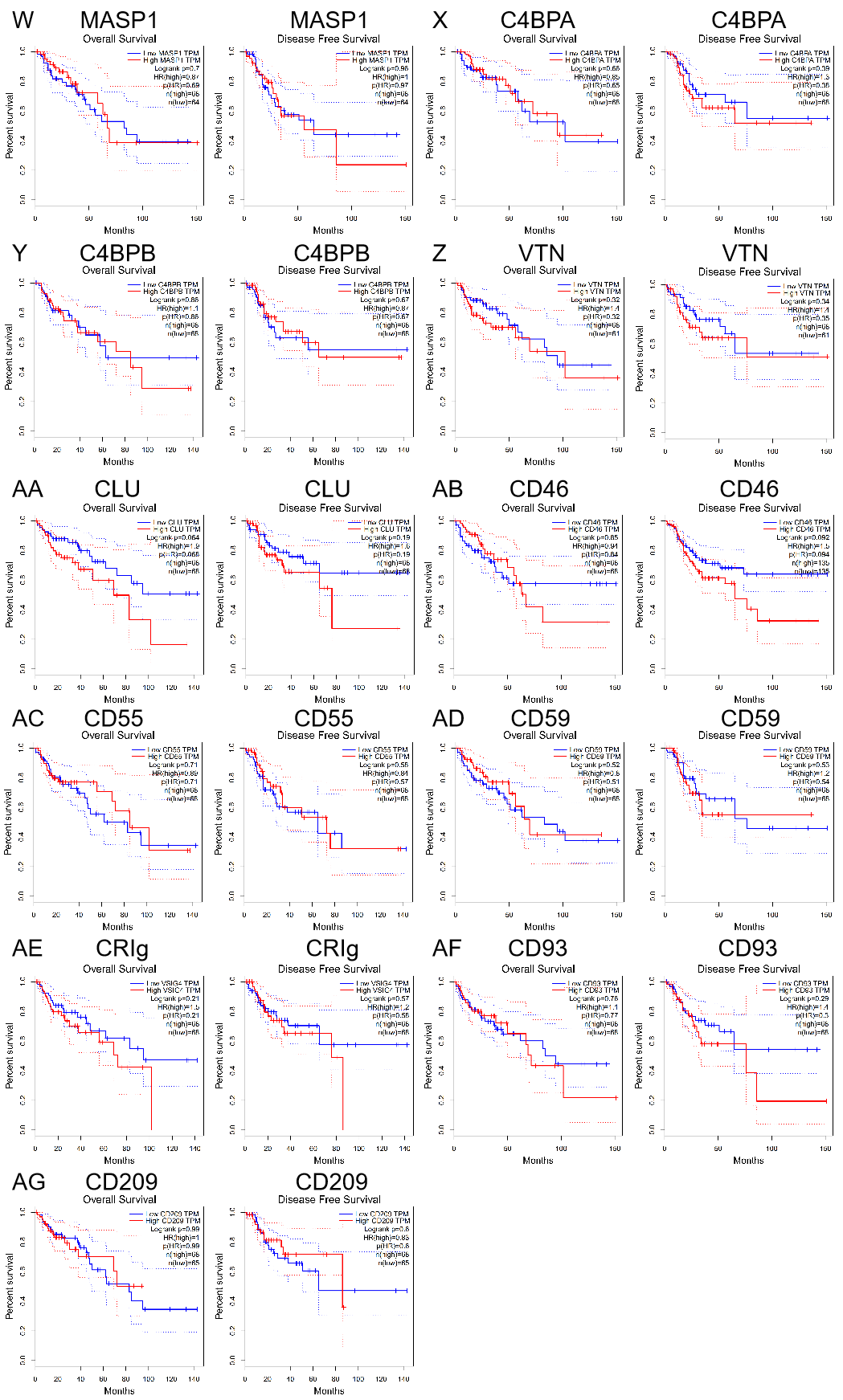


**Supplementary Figure 1.** Correlation of complements expression with prognostic values in COAD. (A) Overall survival and disease free survival curves comparing the high and low expression of C1R. (B) Overall survival and disease free survival curves of C1S. (C) Survival curves of C2. (D) Survival curves of C3. (E) Survival curves of C4. (F) Survival curves of C5 (G) Survival curves of C6. (H) Survival curves of C7. (I) Survival curves of C8G. (J) Survival curves of CFB. (K) Survival curves of CFD. (L) Survival curves of CFF. (M) Survival curves of CFP. (N) Survival curves of CFH. (O) Survival curves of CR1. (P) Survival curves of CR2. (Q) Survival curves of CR3. (R) Survival curves of CR4. (S) Survival curves of C5AR1. (T) Survival curves of C3AR1. (U) Survival curves of C1NH. (V) Survival curves of MASP2. (W) Survival curves of MASP1. (X) Survival curves of C4BPA. (Y) Survival curves of C4BPB. (Z) Survival curves of VTN. (AA) Survival curves of CLU. (AB) Survival curves of CD46. (AC) Survival curves of CD55. (AD) Survival curves of CD59. (AE) Survival curves of CRIg. (AF) Survival curves of CD93. (AG) Survival curves of CD93.

**Supplementary Figure 2.** Correlation of complements expression with prognostic values in STAD (stomach adenocarcinoma).


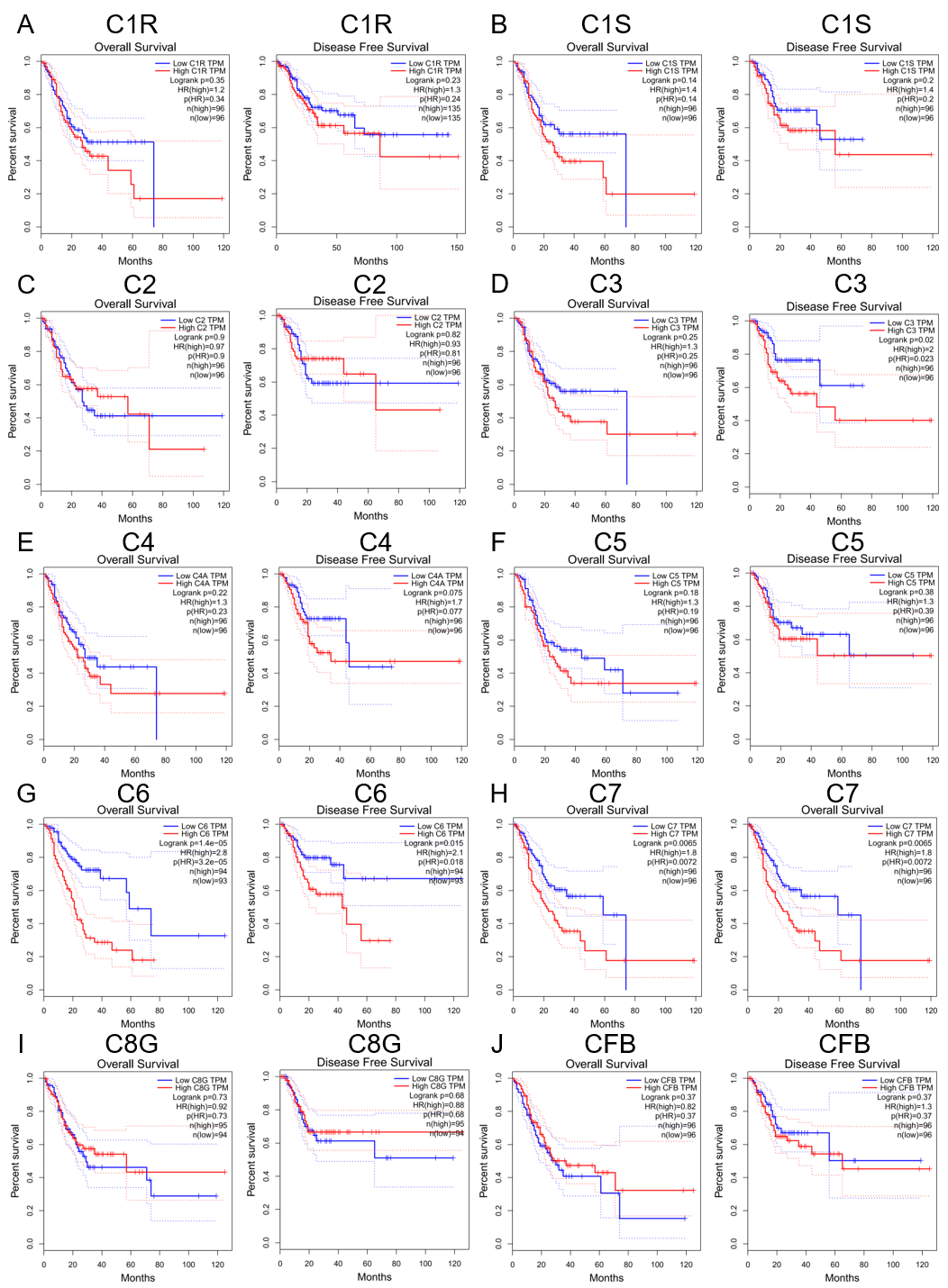

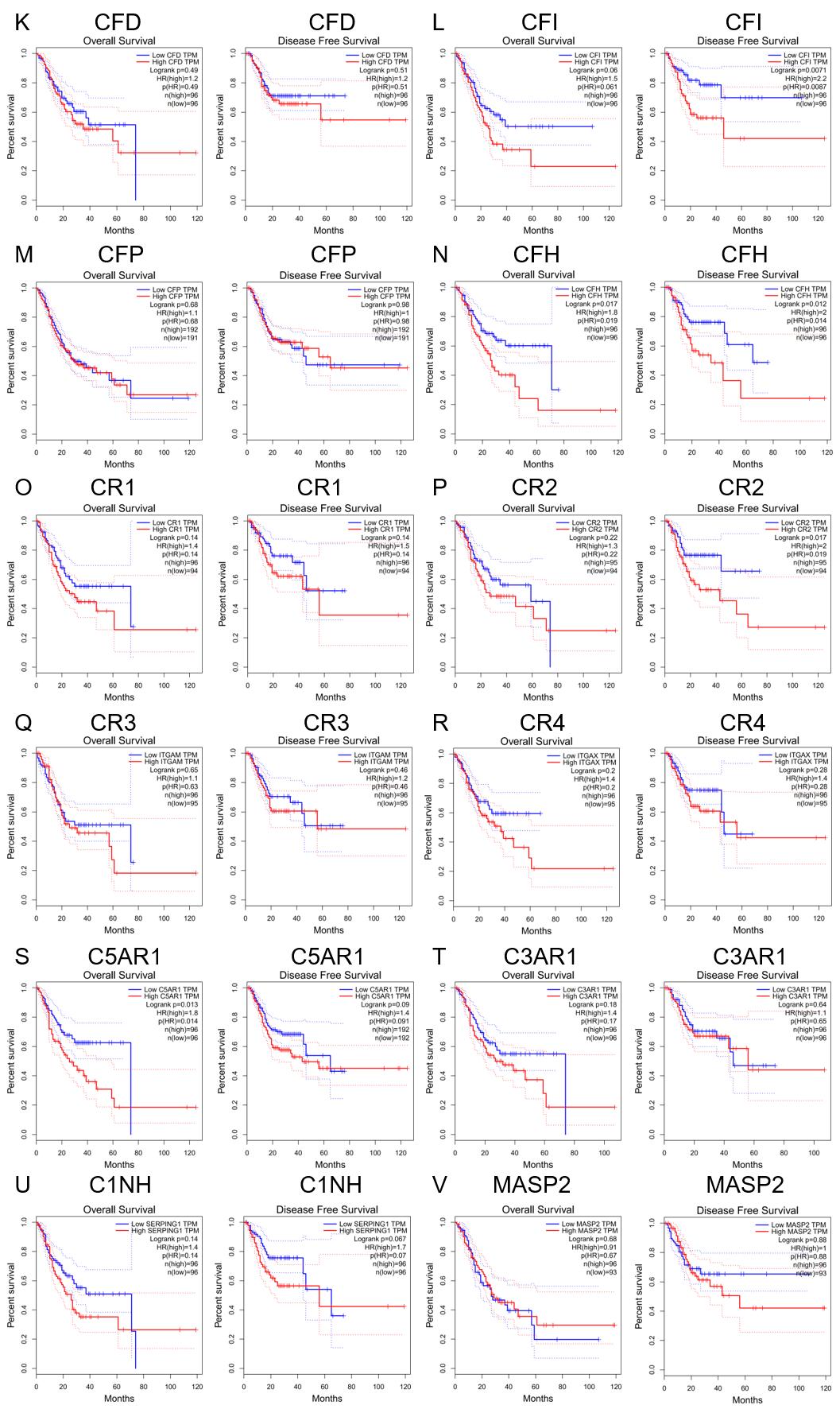

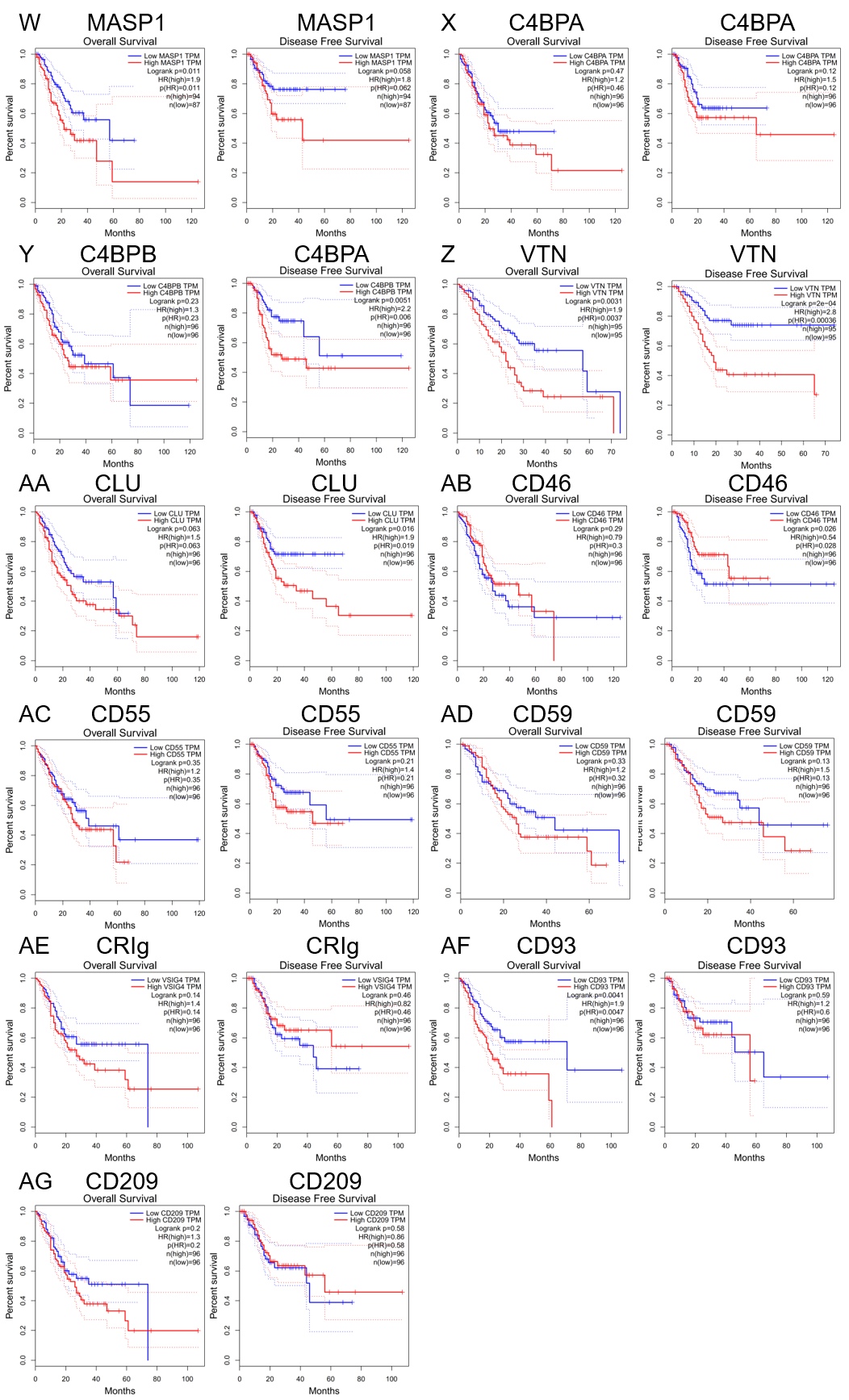


**Supplementary Figure 2.** Correlation of complements expression with prognostic values in STAD. (A) Overall survival and disease free survival curves comparing the high and low expression of C1R. (B) Overall survival and disease free survival curves of C1S. (C) Survival curves of C2. (D) Survival curves of C3. (E) Survival curves of C4. (F) Survival curves of C5 (G) Survival curves of C6. (H) Survival curves of C7. (I) Survival curves of C8G. (J) Survival curves of CFB. (K) Survival curves of CFD. (L) Survival curves of CFF. (M) Survival curves of CFP. (N) Survival curves of CFH. (O) Survival curves of CR1. (P) Survival curves of CR2. (Q) Survival curves of CR3. (R) Survival curves of CR4. (S) Survival curves of C5AR1. (T) Survival curves of C3AR1. (U) Survival curves of C1NH. (V) Survival curves of MASP2. (W) Survival curves of MASP1. (X) Survival curves of C4BPA. (Y) Survival curves of C4BPB. (Z) Survival curves of VTN. (AA) Survival curves of CLU. (AB) Survival curves of CD46. (AC) Survival curves of CD55. (AD) Survival curves of CD59. (AE) Survival curves of CRIg. (AF) Survival curves of CD93. (AG) Survival curves of CD93.

**Supplementary Figure 3.** Correlation of complements expression with immune infiltration level in COAD and STAD via TIMER database
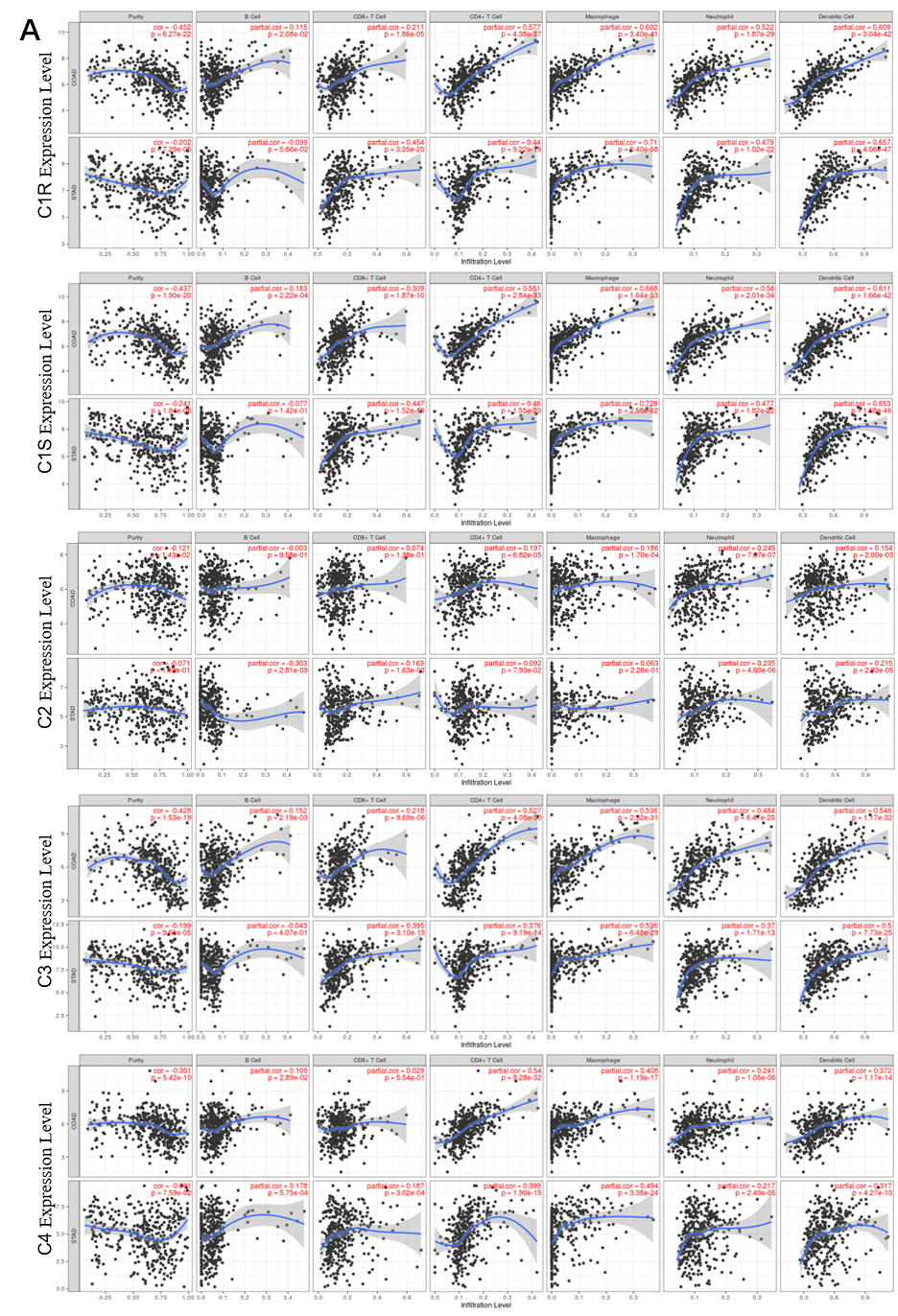

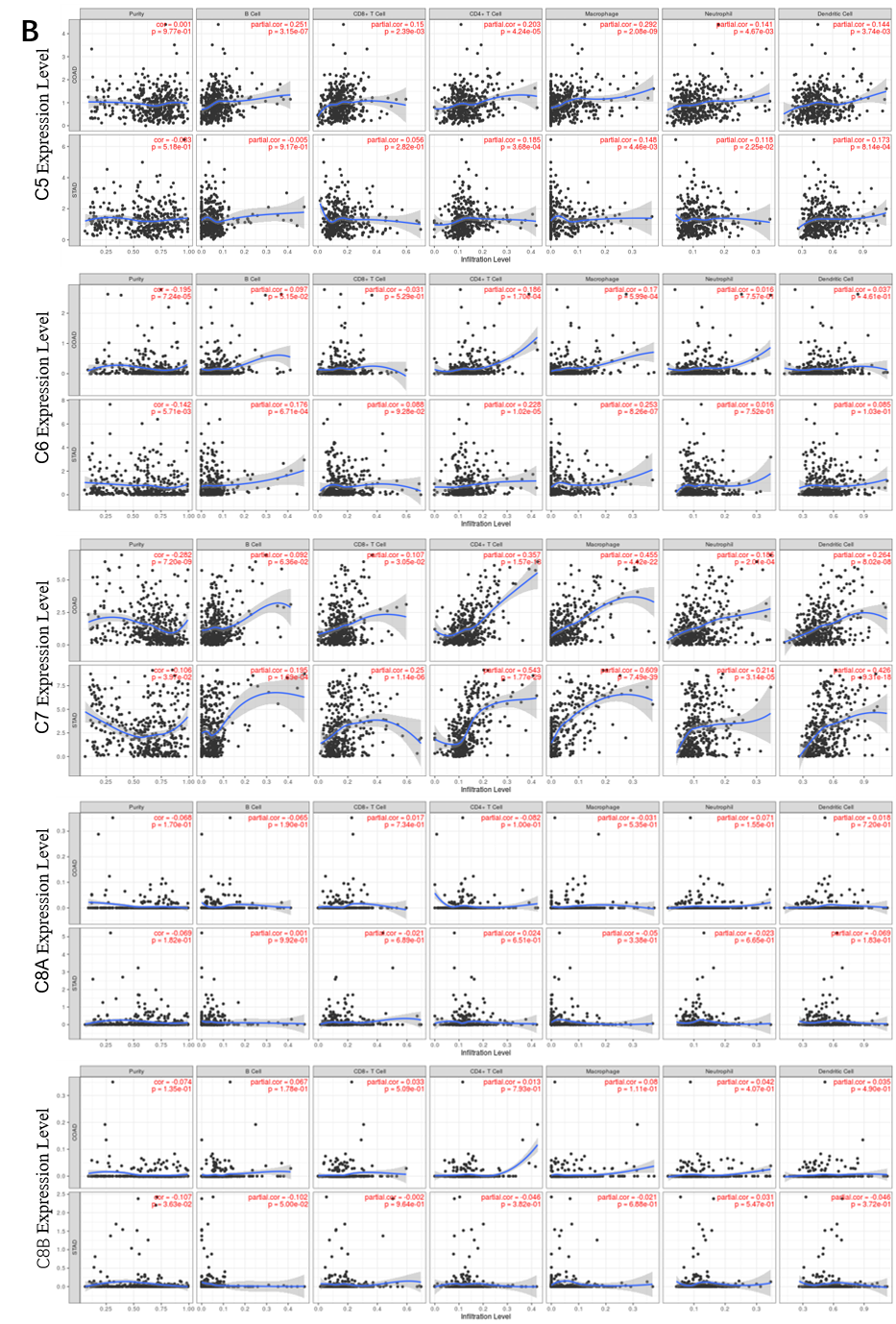

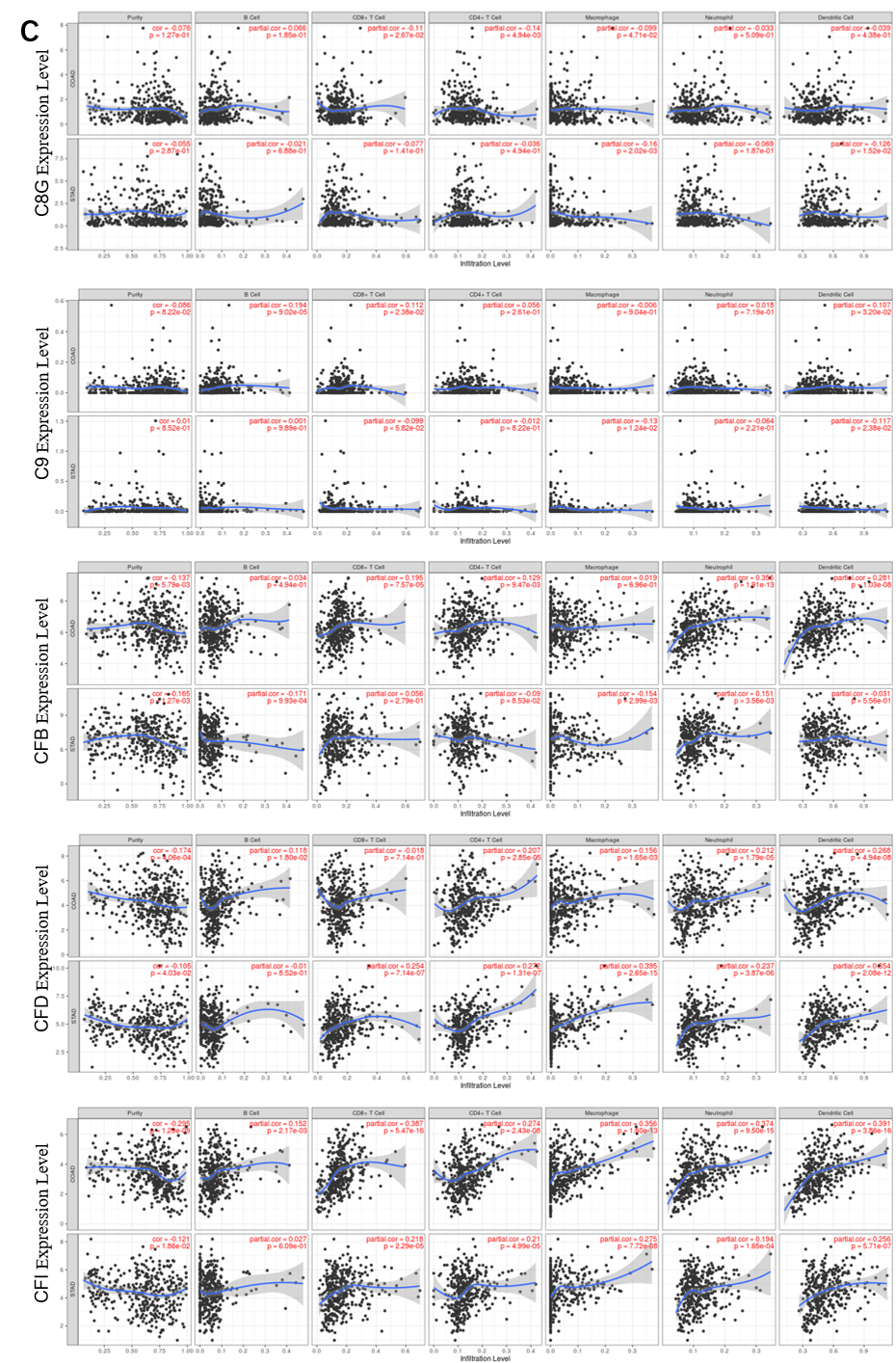


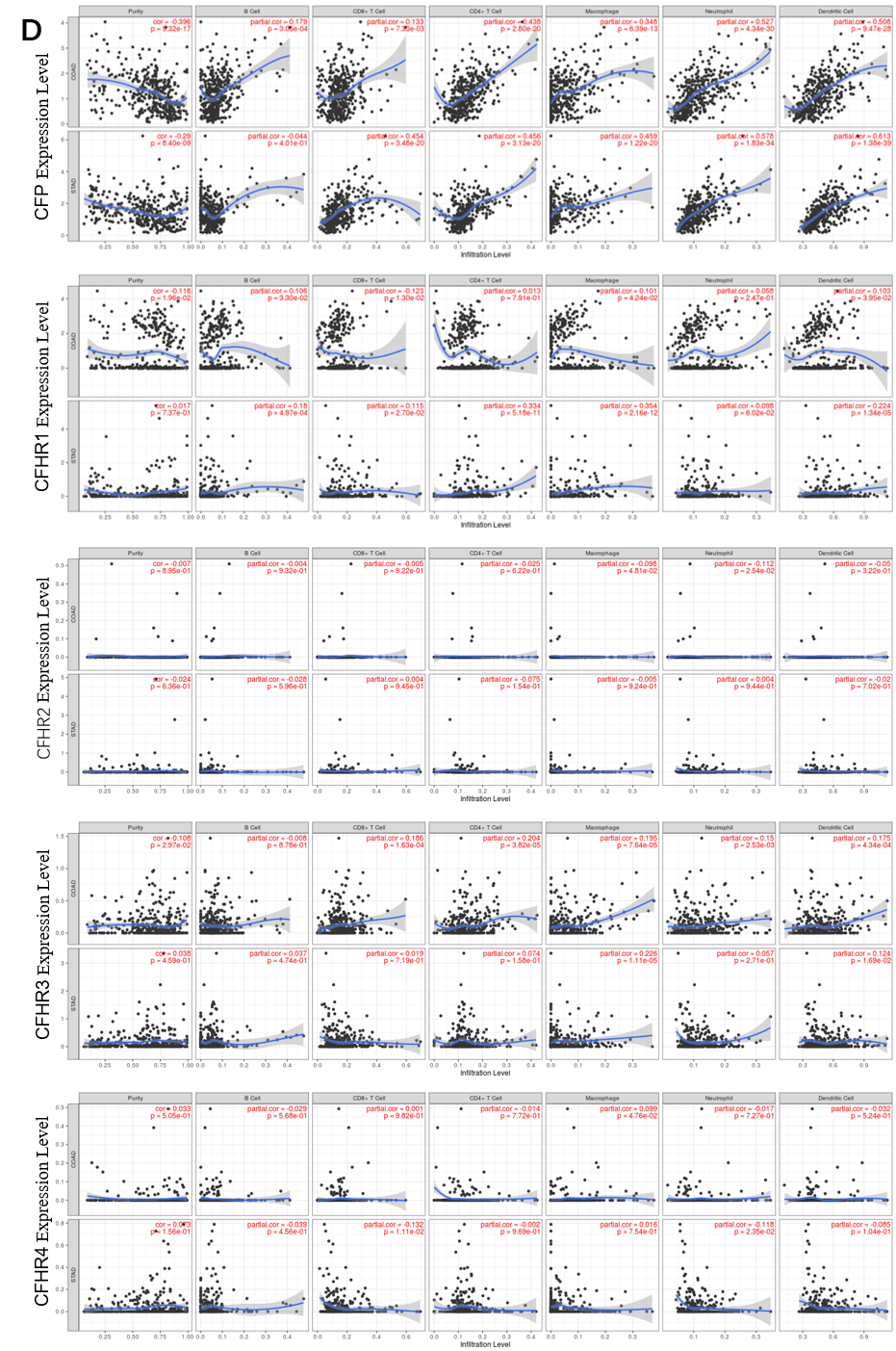

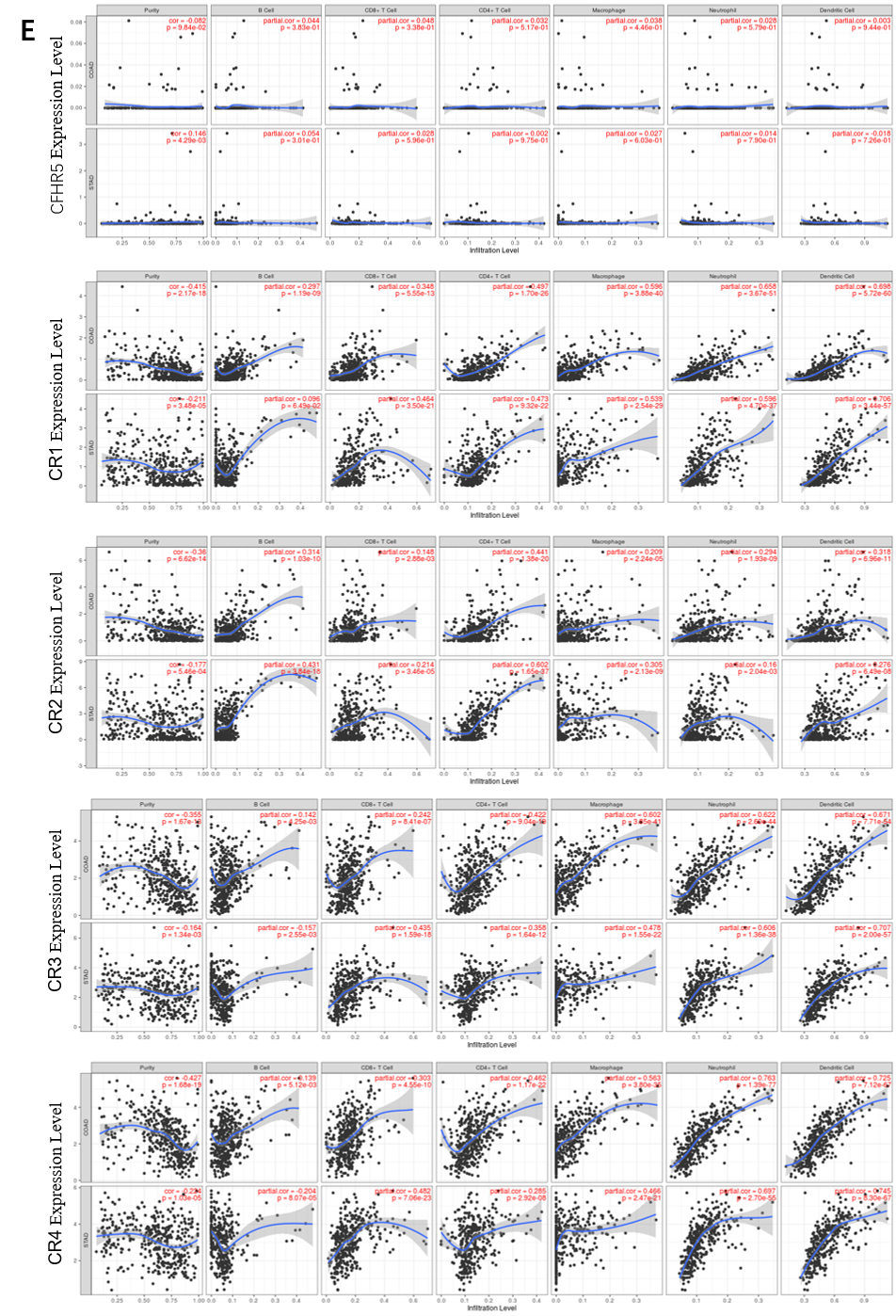

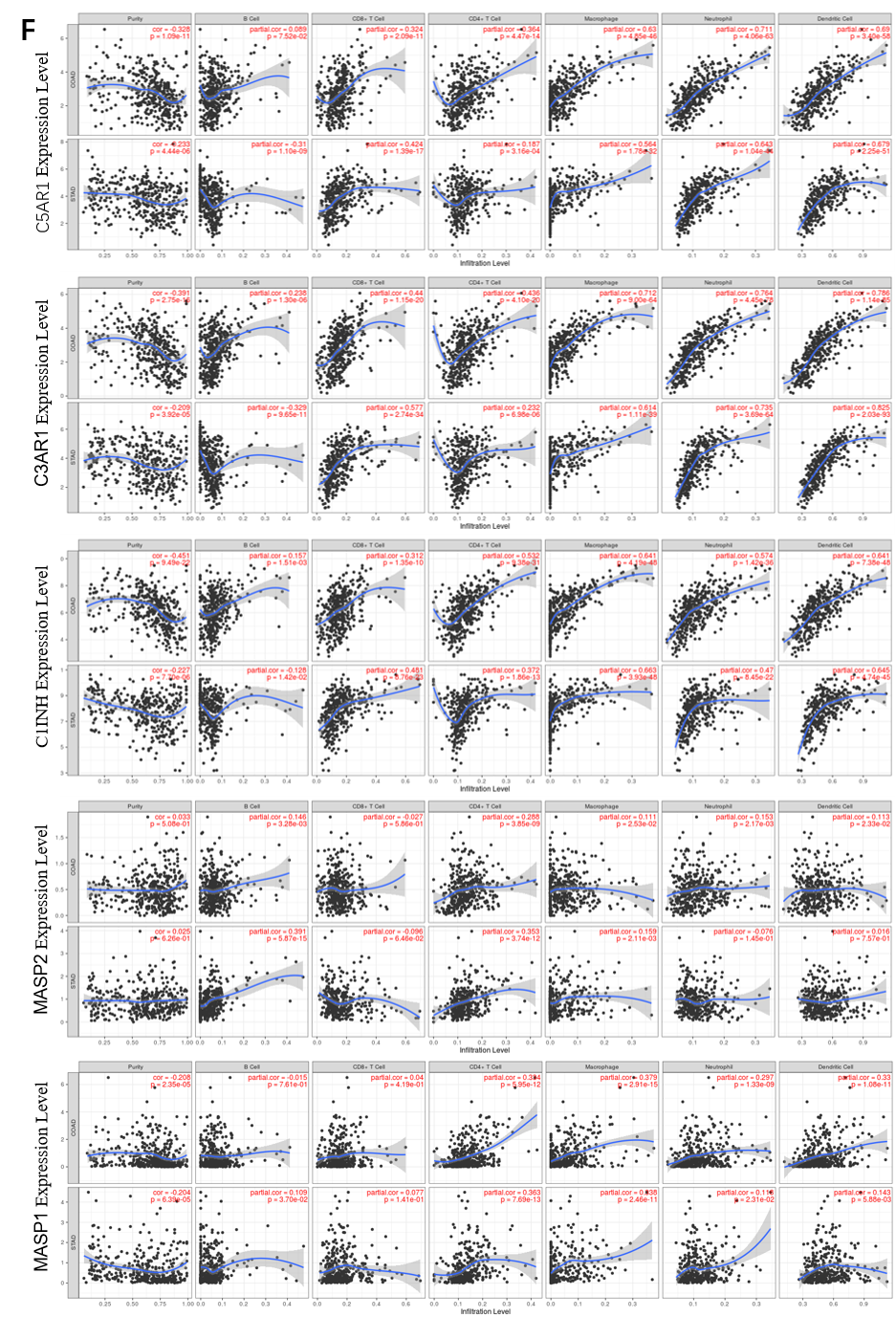


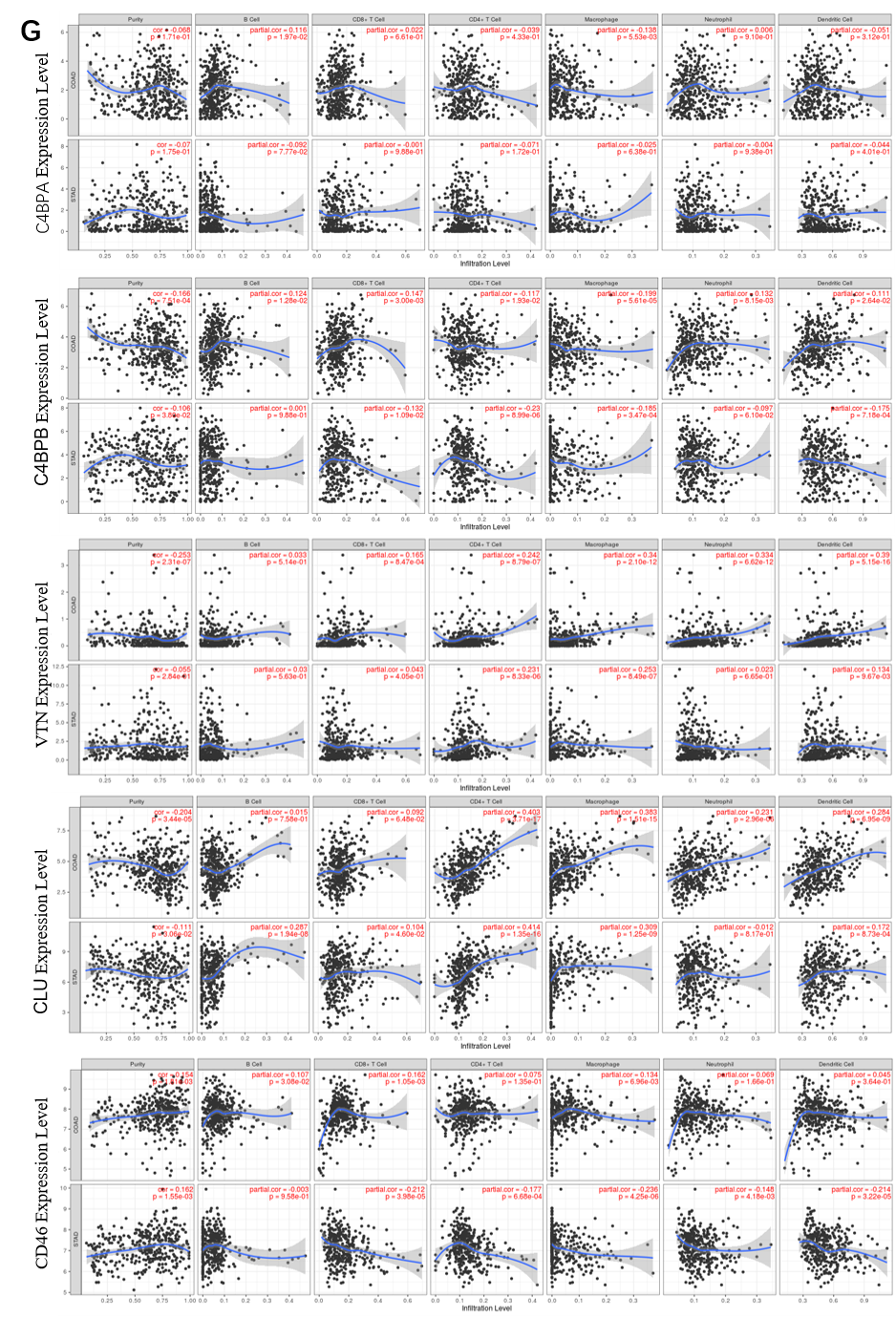

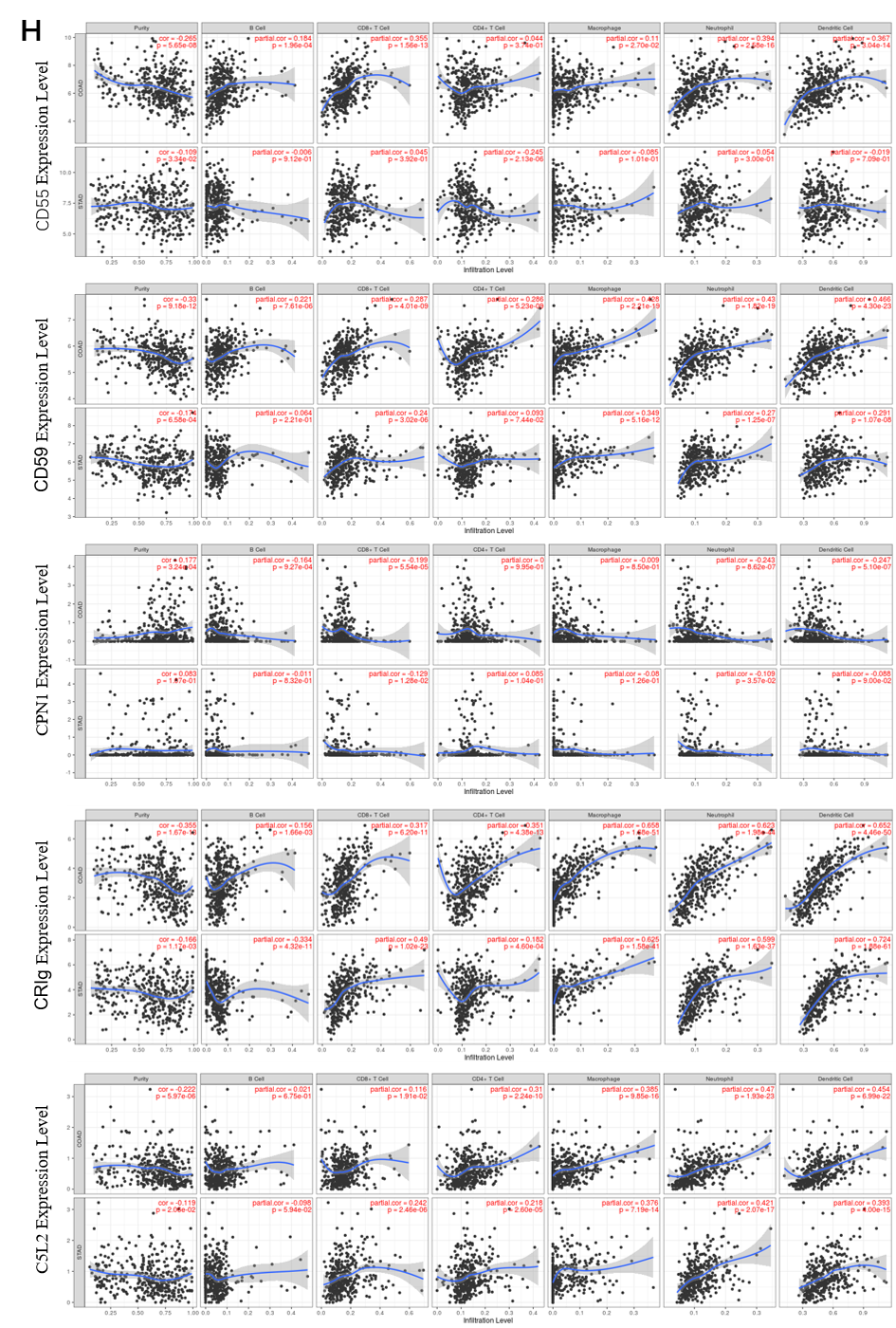

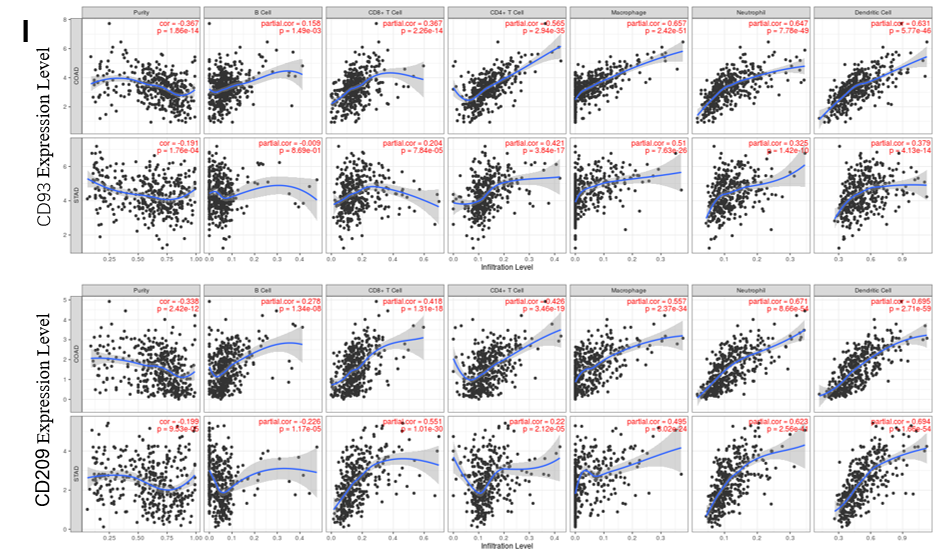


**Supplementary Figure 3.** Correlation of complements expression with immune infiltration levels in COAD and STAD. (A) Correlation of C1R, C1S, C2, C3 and C4 expression with immune infiltration levels in COAD and STAD. (B) Correlation of C5, C5, C7, C8A and C8B expression with immune infiltration levels in COAD and STAD. (C) Correlation of C8G, C9, CFB, CFD, CFI expression with immune infiltration levels in COAD and STAD. (D) Correlation of CFP, CFHR1, CFHR2, CFHR3 and CFHR4 expression with immune infiltration levels in COAD and STAD. (E) Correlation of CFHR5, CR1, CR2, CR3 (ITGAM) and CR4 (ITGAX) expression with immune infiltration levels in COAD and STAD. (F) Correlation of C5AR1, C3AR1, C1NH, MASP2 and MASP1 expression with immune infiltration levels in COAD and STAD. (G) Correlation of C4BPA, C4BPB, VTN, CLU and CD46 expression with immune infiltration levels in COAD and STAD. (H) Correlation of CD55, CD59, CPN1, CRIgand C5L2 expression with immune infiltration levels in COAD and STAD. (I) Correlation of CD93 and CD209 expression with immune infiltration levels in COAD and STAD.

References

1. Gröbner R, K.-S.I., Amberger A, Redolfi R, Dalonneau F, Björck E, Milnes D, Bally I, Rossi V, Thielens N, Stoiber H, Gaboriaud C, Zschocke J., Corrigendum: C1R Mutations Trigger Constitutive Complement 1 Activation in Periodontal Ehlers-Danlos Syndrome. Front Immunol, 2019. 10:2837.doi: 10.3389/fimmu.2019.02837. eCollection 2019.

2. Skerka, P.F.Z.C., Complement regulators and inhibitory proteins. Nat Rev Immunol, 2009 Oct. 2;9(10)729-40.doi: 10.1038/nri2620.

3. Hill A, H.Q., Autoimmune hemolytic anemia. Hematology Am Soc Hematol Educ Program, 2018 Nov. 30;2018(1):382-389.doi: 10.1182/asheducation-2018.1.382.

4. Gómez-Puerta JA, M.C., Vanegas-García AL, Urrego T, Vásquez G, González LA., Anti C1q antibodies. A promising biomarker for cocaine-levamisole induced vasculitis. Reumatol Clin, 2019 Sep - Oct. 15(5):e66-e67.doi: 10.1016/j.reuma.

5. Patol, V.V.G.A.C.N.G.E.A., The clinical and morphological characteristics of C1q glomerulopathy. 80（1）：46-51, 2018.doi: 10.17116 / patol201880146-51.

6. Bulla R, T.C., Rami D, et al, C1q acts in the tumour microenvironment as a cancer-promoting factor independently of complement activation. Nat Commun, 2016. 7:10346.doi: 10.1038/ncomms10346.

7. Chen HH, T.L., Lee KR, Chen YM, Hung WT, Chen DY, Genetic association of complement component 2 polymorphism with systemic lupus erythematosus. Tissue Antigens, 2015 Aug. 86(2):122-33.doi: 10.1111/tan.12602.

8. Ajona D, O.-E.S., Pio R, Complement anaphylatoxins C3a and C5a: emerging roles in cancer progression and treatment. Semin Cell Dev Biol, 2019. 85:153–163.doi: 10.1016/j.semcdb.2017.11.023.

9. Yu Z, O.C., Aiba S, et al, Therapeutic concentration of lithium stimulates complement C3 production in dendritic cells and microglia via GSK-3 inhibition. Glia, 2015. 63(2):257–270.doi: 10.1002/glia.22749.

10. Boire A, Z.Y., Shieh J, et al. , Complement component 3 adapts the cerebrospinal fluid for leptomeningeal metastasis. Cell, 2017. 168(6):1101–1113.e1113.doi: 10.1016/j.cell.2017.02.025.

11. Ajona D, P.M., Corrales L, et al, Investigation of complement activation product c4d as a diagnostic and prognostic biomarker for lung cancer. J Natl Cancer Inst, 2013. 105(18):1385–1393.doi: 10.1093/jnci/djt205.

12. Ajona D, P.M., Chiara MD, et al, Complement activation product C4d in oral and oropharyngeal squamous cell carcinoma. Oral Dis, 2015. 21(7):899–904.doi: 10.1111/odi.12363.

13. Vadrevu SK, C.N., Sharma SK, Sharma P, Cleveland C, Riediger L, Manne S, Fairlie DP, Gorczyca W, Almanza O, Karbowniczek M, Markiewski MM, Complement c5a receptor facilitates cancer metastasis by altering T-cell responses in the metastatic niche. Cancer Res, 2014. 74(13):3454–3465.doi: 10.1158/0008-5472.

14. Piao C, C.L., Qiu S, et al, Complement 5a enhances hepatic metastases of colon cancer via monocyte chemoattractant protein-1-mediated inflammatory cell infiltration. J Biol Chem, 2015. 290(17):10667–10676.doi: 10.1074/jbc.M114.612622.

15. Corrales L, A.D., Rafail S, Lasarte JJ, Riezu-Boj JI, Lambris JD, Rouzaut A, Pajares MJ, Montuenga LM, Pio R, Anaphylatoxin C5a creates a favorable microenvironment for lung cancer progression. J Immunol, 2012. 189(9):4674–4683.doi: 10.4049/jimmunol.1201654.

16. Hu WH, H.Z., Shen X, et al, C5a receptor enhances hepatocellular carcinoma cell invasiveness via activating ERK1/2-mediated epithelial–mesenchymal transition. Exp Mol Pathol, 2016. 100(1):101–108.doi: 10.1016/j.yexmp.2015.10.001.

17. Chen J, L.G., Zhang L, et al, Complement C5a/C5aR pathway potentiates the pathogenesis of gastric cancer by down-regulating p21 expression. Cancer Lett, 2018. 412:30–36.doi: 10.1016/j.canlet.2017.10.003.

18. Keyvan Rad J, M.A., Khoei S, Shirvalilou S, Enhanced Photogeneration of Reactive Oxygen Species and Targeted Photothermal Therapy of C6 Glioma Brain Cancer Cells by Folate-Conjugated Gold-Photoactive Polymer Nanoparticles. ACS Appl Mater Interfaces, 2018 Jun 13;10(23):19483-19493.doi: 10.1021/acsami.8b05252.

19. Seol HS, L.S., Song JS, et al, Complement proteins C7 and CFH control the stemness of liver cancer cells via LSF-1. Cancer Lett, 2016. 372(1):24–35.doi: 10.1016/j.canlet.2015.12.005.

20. Bubeck D, R.P., Donev R, Morgan BP, Llorca O, Lea SM., Structure of human complement C8, a precursor to membrane attack. J Mol Biol, 2011 Jan 14;405(2):325-30.doi: 10.1016/j.jmb.2010.10.031.

21. Lovelace LL, C.C., Sodetz JM, Lebioda L, Structure of human C8 protein provides mechanistic insight into membrane pore formation by complement. J Biol Chem, 2011 May. 20;286(20):17585-92.doi: 10.1074/jbc.M111.219766.

22. Kremlitzka M, e.a., Functional analyses of rare genetic variants in complement component C9 identified in patients with age-related macular degeneration. Hum Mol Genet, 2018 Aug. 1;27(15):2678-2688.doi: 10.1093/hmg/ddy178.

23. function, H.C.v.s.C.c.s.a.i.m.a.c., Hepatitis C virus suppresses C9 complement synthesis and impairs membrane attack complex function. J Virol, 2013 May. 87(10):5858-67.doi: 10.1128/JVI.00174-13.

24. Longhurst H, e.a., Prevention of hereditary angioedema attacks with a subcutaneous C1 inhibitor. N Engl J Med, 2017. 376:1131–1140.

25. Davis, A.E., Mejia, P. Lu, F, Biological activities of C1 inhibitor. Mol. Immunol, 2008. 45(16):4057-63.

26. Degn SE, e.a., MAp19, the alternative splice product of the MASP2 gene. J Immunol Methods, 2011. 373:89–101.

27. Degn SE, e.a., MAp44, a human protein associated with pattern recognition molecules of the complement system and regulating the lectin pathway of complement activation. J Immunol., 2009. 183:7371–7378.

28. Rooryck C, e.a., Mutations in lectin complement pathway genes COLEC11 and MASP1 cause 3MC syndrome. Nat Genet, 2011. 43:197–203.

29. Blom AM, e.a., A novel non-synonymous polymorphism (pArg240His) in C4b-binding protein is associated with atypical hemolytic uremic syndrome and leads to impaired alternative pathway cofactor activity. J Immunol., 2008. 180:6385–6391.

30. Mohlin FC, e.a., Analysis of genes coding for CD46, CD55, and C4b-binding protein in patients with idiopathic, recurrent, spontaneous pregnancy loss. Eur J Immunol, 2013. 43:1617–1629.

31. Klein RJ, e.a., Complement factor H polymorphism in age-related macular degeneration. Science., 2005. 308:385–389.

32. Edwards AO, e.a., Complement factor H polymorphism and age-related macular degeneration. Science., 2005. 308:421–424.

33. Abrera-Abeleda MA, e.a., Variations in the complement regulatory genes factor H (CFH) and factor H related 5 (CFHR5) are associated with membranoproliferative glomerulonephritis type II (dense deposit disease). J Med Genet, 2006. 43:582–589.

34. Bernabeu-Herrero ME, e.a., Complement factor H, FHR-3 and FHR-1 variants associate in an extended haplotype conferring increased risk of atypical hemolytic uremic syndrome. Mol Immunol, 2015. 67:276–286.

35. Vernon KA, e.a., Partial complement factor H deficiency associates with C3 glomerulopathy and thrombotic microangiopathy. J Am Soc Nephrol., 2016. 27:1334–1342.

36. Reis ES, F.D., Isaac L, Clinical aspects and molecular basis of primary deficiencies of complement component C3 and its regulatory proteins factor I and factor H. Scand J Immunol, 2006. 63:155–168.

37. Clark SJ, e.a., Identification of factor H-like protein 1 as the predominant complement regulator in Bruch’s membrane: implications for age-related macular degeneration. J Immunol, 2014. 193:4962–4970.

38. Hakobyan S, T.A., Harris CL, de Cordoba SR, Morgan BP, Variant-specific quantification of factor H in plasma identifies null alleles associated with atypical hemolytic uremic syndrome. Kidney Int, 2010. 78:782–788.

39. Kim SH, L.M., Hwang HK, Lee SH, Kim H, Paik YK, Kang CM., Prognostic potential of the preoperative plasma complement factor B in resected pancreatic cancer: A pilot study. Cancer Biomark, 2019. 24(3):335-342.doi: 10.3233/CBM-181847.

40. Riihilä P, N.L., Farshchian M, Kallajoki M, Kivisaari A, Meri S, Grénman R, Peltonen S, Peltonen J, Pihlajaniemi T, Heljasvaara R, Kähäri VM, Complement Component C3 and Complement Factor B Promote Growth of Cutaneous Squamous Cell Carcinoma. Am J Pathol, 2017May. 187(5):1186-1197.

41. Bresin E, e.a., Combined complement gene mutations in atypical hemolytic uremic syndrome influence clinical phenotype. J Am Soc Nephrol, 2013. 24:475–486.

42. van de Ven JP, e.a., A functional variant in the CFI gene confers a high risk of age-related macular degeneration. Nat Genet, 2013. 45:813–817.

43. Schwarz, M.e.a., Potential protective role of apoprotein J (clusterin) in atherogenesis: binding to enzymatically modified low-density lipoprotein reduces fatty acid-mediated cytotoxicity. . Thromb. Haemost, 2008. 100, 110–118.

44. McDonald JF, N.G., Potent inhibition of terminal complement assembly by clusterin: characterization of its impact on C9 polymerization. Biochemistry, 1997. 36:7464–7473.

45. Nomura M, e.a., Genomic analysis of idiopathic infertile patients with sperm-specific depletion of CD46. . . Exp Clin Immunogenet, 2001. 18:42–50.

46. Leung TH, T.H., Siu MK, Chan DW, Chan KK, Cheung AN, Ngan HY., Human papillomavirus E6 protein enriches the CD55(+) population in cervical cancer cells, promoting radioresistance and cancer aggressiveness. J Pathol, 2018. 244(2):151-163.doi: 10.1002/path.4991.

47. He Z, W.H., Jiao Y, Zheng J, Expression and prognostic value of CD97 and its ligand CD55 in pancreatic cancer. Oncol Lett, 2015 Feb. 9(2):793-797.

48. Ozen A, C.W., Ardy RC, Domínguez Conde C. et al, CD55 Deficiency, Early-Onset Protein-Losing Enteropathy, and Thrombosis. N Engl J Med, 2017 6;377(1):52-61.doi: 10.1056/NEJMoa1615887.

49. Ueda M, e.a., . Endovascular trophoblast expresses CD59 to evade complement-dependent cytotoxicity. Mol Cell Endocrinol, 2019 Jun 15. 490:57-67.doi: 10.1016/j.mce.2019.04.006.

50. Wilcox LA, E.J., Bernshaw NJ, Parker CJ, Molecular basis of the enhanced susceptibility of the erythrocytes of paroxysmal nocturnal hemoglobinuria to hemolysis in acidified serum. Blood, 1991. 78:820–829.

51. Nevo Y, e.a., CD59 deficiency is associated with chronic hemolysis and childhood relapsing immune-mediated polyneuropathy. Blood, 2013. 121:129–135.

52. Zhou Y, C.L., Wang Q, et al, CD59 is a potential biomarker of esophageal squamous cell carcinoma radioresistance by affecting DNA repair. Cell Death Dis, 2018 Aug 30. 9(9):887.

53. Cui R, Z.P., Li Y, Role of Carboxypeptidase N Invasion and Migration in Breast Cancer. Anticancer Agents Med Chem, 2016. 16(9):1198-202.

54. Sigler C, A.K., Cooper K, Haber H, Van deCarr S, Examination of baseline levels of carboxypeptidase N and complement components as potential predictors of angioedema associated with the use of an angiotensin-converting enzyme inhibitor. Arch Dermatol, 1997. 133:972–975.

55. Mathews KP, P.P., Amendola MA, Lewis FH. , Plasma protease inhibitor and anaphylatoxin inactivator levels in chronic urticaria/angioedema and in patients experiencing anaphylactoid reactions to radiographic contrast media. Int Arch Allergy Appl Immunol, 1986. 79:220–223.

56. Zhu XC, Y.J., Jiang T, Wang P, Cao L, Tan L, CR1 in Alzheimer's disease. Mol Neurobiol, 2015 Apr. 51(2):753-65.doi: 10.1007/s12035-014-8723-8.

57. Ross GD, e.a., Disease-associated loss of erythrocyte complement receptors (CR1, C3b receptors) in patients with systemic lupus erythematosus and other diseases involving autoantibodies and/or complement activation. J Immunol, 1985.doi: 135:2005–2014.

58. Rondelli T, e.a., Polymorphism of the complement receptor 1 gene correlates with the hematologic response to eculizumab in patients with paroxysmal nocturnal hemoglobinuria. Haematologica, 2014. 99:262–266.

59. Cockburn IA, e.a., A human complement receptor 1 polymorphism that reduces Plasmodium falciparum rosetting confers protection against severe malaria. Proc Natl Acad Sci USA, 2004. 101:272–277.

60. Kim TH, B.S., Lee SH, Kim SY, Baek SH., Association of Complement Receptor 2 Gene Polymorphisms with Susceptibility to Osteonecrosis of the Femoral Head in Systemic Lupus Erythematosus. Biomed Res Int, 2016. 9208035.doi: 10.1155/2016/9208035.

61. Khandelwal S, e.a., The antigenic complex in HIT binds to B cells via complement and complement receptor 2 (CD21). Blood, 2016 Oct 6. 128(14):1789-1799.

62. Rahkola D, L.J., Siiskonen H, Pelkonen J, Harvima IT, Mast Cells Are a Marked Source for Complement C3 Products That Associate with Increased CD11b-Positive Cells in Keratinocyte Skin Carcinomas. Cancer Invest, 2019. 37(2):73-84.doi: 10.1080/07357907.2019.

63. Li C, T.F., Ma Y, Qian K, Zhang J, Chen X, Association of the CD11b rs1143679 polymorphism with systemic lupus erythematosus in the Han Chinese population. J Int Med Res, 2018 Mar. 46(3):1008-1014.

64. Lee H, L.H., Song IH, Bang WS, Heo SH, Gong G, Park IA, CD11c-Positive Dendritic Cells in Triple-negative Breast Cancer. In Vivo, 2018 32(6):1561-1569.doi: 10.21873/invivo.11415.

65. Umit EG, e.a., CD11c expression in chronic lymphocytic leukemia revisited, related with complications and survival. Int J Lab Hematol, 2017 Oct. 39(5):552-556.doi: 10.1111/ijlh.12695.

66. Park YJ, P.M., Park S, Lee ES, CD11c is upregulated in CD8+ T cells of patients with Behçet's disease. Clin Exp Rheumatol, 2016 Sep-Oct. 34(6 Suppl 102):S86-S91.

67. Wang Y, X.B., et al, High expression of CD11c indicates favorable prognosis in patients with gastric cancer. World J Gastroenterol, 2015 Aug 21. 21(31):9403-12.

68. Byun JM, J.D., Choi IH, Lee DS, Kang MS, The Significance of VSIG4 Expression in Ovarian Cancer. Int J Gynecol Cancer, 2017 Jun. 27(5):872-878.doi: 10.1097/IGC.0000000000000979.

69. Textoris J, I.D., Ben Amara A, Evaluation of current and new biomarkers in severe preeclampsia: a microarray approach reveals the VSIG4 gene as a potential blood biomarker. PLoS One, 2013 Dec 9. 8(12):e82638.doi: 10.1371/journal.pone.0082638.

70. He, J.Q., Wiesmann, C.van Lookeren Campagne, M, A role of macrophage complement receptor CRIg in immune clearance and inflammation. Mol. Immunol, 2008. 45, 4041–4047.

71. Ward, P.A., Functions of C5a receptors. J. Mol. Med, 2009. 87, 375–378.

72. Scola, A.M., Johswich, K. O., Morgan, B. P., Klos, A. Monk, P. N, The human complement fragment receptor, C5L2, is a recycling decoy receptor. Mol. Immunol, 2009. 46, 1149–1162.

73. Rittirsch, D.e.a., Functional roles for C5a receptors in sepsis. Nature Med, 2008. 14, 551–557.

74. Nabizadeh JA, M.H., Panagides N, Steyn FJ, Lee JD, Li XX, Akhir FNM, Chen W, Boyle GM, Taylor SM, Woodruff TM, Rolfe BE, C5a receptors C5aR1 and C5aR2 mediate opposing pathologies in a mouse model of melanoma. FASEB J, 2019 Oct. 33(10):11060-11071.doi: 10.1096/fj.201800980RR.

75. Benard, M.e.a., Role of complement anaphylatoxin receptors (C3aR, C5aR) in the development of the rat cerebellum. Mol. Immunol, 2008. 45, 3767–3774.

76. Yu Wang, H.Z., and You-Wen He, The Complement Receptors C3aR and C5aR Are a New Class of Immune Checkpoint Receptor in Cancer Immunotherapy. Front Immunol, 2019 Jul. 10: 1574.doi: 10.3389/fimmu.2019.01574.

77. Köhl, J.e.a.J.C.I., A regulatory role for the C5a anaphylatoxin in type 2 immunity in asthma. 116, 783–796, 2006.

78. Li K, W.K., Wu W, Wang N, Zhang T, Choudhry N, Song Y, Farrar CA, Ma L, Wei LL, Duan ZY, Dong X, Liu EQ, Li ZF, Sacks SH, Zhou W., C5aR1 promotes acute pyelonephritis induced by uropathogenic E. coli. JCI Insight, 2017 Dec 21. 2(24).doi: 10.1172/jci.insight.97626.

79. Tosi GM, e.a., CD93 as a Potential Target in Neovascular Age-Related Macular Degeneration. J Cell Physiol, 2017 Jul 232(7):1767-1773.doi: 10.1002/jcp.25689.

80. Duvetorp A, e.a., Psoriasis and Pro-angiogenetic Factor CD93: Gene Expression and Association with Gene Polymorphism Suggests a Role in Disease Pathogenesis. Acta Derm Venereol, 2017 Aug 31. 97(8):916-921.doi: 10.2340/00015555-2682.

81. Park HJ, O.E., Han HJ, Park KH, Jeong KY, Park JW, Lee JH, Soluble CD93 in allergic asthma. Sci Rep, 2020 Jan 15. 10(1):323.doi: 10.1038/s41598-019-57176-2.

82. Yi L, Z.K., Mo Y, Zhen G, Zhao J, The association between CD209 gene polymorphisms and pulmonary tuberculosis susceptibility: a meta-analysis. Int J Clin Exp Pathol, 2015 Oct 1. 8(10):12437-45.

83. He YX, Y.C., Zhang P, Li Q, Park CG, et al, Yersinia pseudotuberculosis Exploits CD209 Receptors for Promoting Host Dissemination and Infectio. Infect Immun, 2018 Dec 19. 87(1).doi: 10.1128/IAI.00654-18.

84. Choi B, S.C., Kim HA, Sayeed HM, Sohn S, The Correlation of CD206, CD209, and Disease Severity in Behçet's Disease with Arthritis. Mediators Inflamm, 2017. 2017:7539529.doi: 10.1155/2017/7539529.
